# Autonomous and Communicative Microcapsule Systems for Life‐Like Homeostatic pH Regulation

**DOI:** 10.1002/smll.73756

**Published:** 2026-05-13

**Authors:** Hongda Zhou, James Smith, Rui Cheng, Ramzan Ullah, Huaiyuan Wang, Dmitry Shchukin

**Affiliations:** ^1^ School of Chemical Engineering and Technology and State Key Laboratory of Chemical Engineering and Low‐Carbon Technology Tianjin University Tianjin P. R. China; ^2^ Stephenson Institute for Renewable Energy University of Liverpool Liverpool UK; ^3^ Gulliver CNRS UMR 7083 ESPCI Paris PSL Research University Paris France

**Keywords:** 3D‐printed microfluidic, feedback, homeostasis, pH regulation, responsive microcapsule

## Abstract

Life sustains complex functions through intercellular communication networks that coordinate collective behavior and regulate environmental homeostasis. Replicating such dynamic and autonomous control over extended spatial and temporal domains in synthetic materials remains significant challenges. Here, we present a programmable dual‐microcapsule system that emulates life‐like homeostatic pH regulation via an antagonistic enzymatic network. The system integrates urease microcapsules (UMCs) and esterase microcapsules (EMCs), which were produced using microfluidic devices coupled with a surface co‐assembly strategy. By integrating hybrid junction geometry with a modified epoxy post‐coating strategy, the 3D‐printed microfluidic device overcomes intrinsic structural defects and surface irregularities. The resulting amphiphobic and defect‐free channels ensure stable droplet production and precise microsphere fabrication. Individual capsules display pH‐mediated negative feedback through adaptive shell permeability, whereas mixed populations display communicate behaviors via pH signaling to generate programmable pH oscillations and feedback‐controlled pH stabilization. This platform exhibits robust reaction to external pH control, long‐term cycling stability, and inter‐capsule interaction. This work offers a versatile route for engineering communicative, autonomous, and adaptive material systems, with broad implications for biomedical devices, environmental regulation, and soft control using chemical information exchange.

## Graphical abstract

A dual‐microcapsule system mimicking biological homeostasis is developed, enabling programmable pH oscillations through antagonistic enzymatic feedback. Monodisperse urease and esterase capsules, produced via hybrid‐junction 3D‐printed microfluidics with epoxy‐modified channels, exhibit adaptive shell permeability and inter‐capsule communication. The platform offers a versatile strategy for constructing autonomous, communicative, and environmentally responsive soft materials.

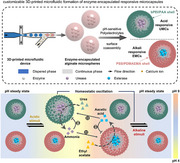

## 1 Introduction

The orchestrated capacity to sustain internal equilibrium amid external perturbations, referred to as homeostasis, is a defining characteristic of biological systems [1, 2]. As a central parameter shaping cellular microenvironments, pH control governs diverse biological processes such as metabolic pathways, osteogenic remodeling, tumor microenvironmental modulation, and intestinal microbial homeostasis [3, 4]. One prominent example is that tumor cells leverage acidity to subvert immunity, eliciting countermeasures by immune cells through metabolic adaptation and localized alkalization [5]. These homeostatic regulations are governed by complex, out‐of‐equilibrium biochemical processes involving continuous kinetic control, energy dissipation, and tightly feedback loops [6, 7, 8]. Despite advances in dissipative and feedback‐controlled systems such as chemomechanical feedback and physical/chemical oscillator, the capacity to emulate life‐like homeostatic regulation over extended temporal and spatial domains remains a considerable challenge [9, 10, 11]. Reconfiguring these principles within synthetic systems to embed stimuli‐responsive, signal transduction, homeostasis, and programmed regulation under out‐of‐equilibrium conditions is instrumental in the design of interactive materials or systems [12, 13, 14]. Multicellular communication serves as the “operating system” for the dynamic balance of the microenvironment [15, 16]. By mimicking its control logic (such as pH oscillation thresholds), targeted reprogramming strategies for the microenvironment can be developed.

Microcapsules with core–shell architectures can mimic organelle‐like compartments, enabling dynamic modulation of shell permeability to small molecules under external chemical or physical stimuli [17, 18, 19]. Such capabilities make them perspective candidates for constructing adaptive synthetic systems, enabling enzyme‐loaded nanoreactors that operate exclusively under out‐of‐equilibrium conditions to achieve homeostatic oscillations and programmable regulation [20, 21, 22]. While biological systems exploit membrane transporters, feedback communication, and enzymatic cascades, synthetic counterparts could utilize pH‐responsive enzymatic circuits (e.g., urea–urease, ester–esterase) to achieve autonomous regulation [23, 24, 25, 26]. Recent progress has focused on individual microcapsule behaviors (e.g., stimuli‐responsive, controlled microreactors), yet assembling heterogeneous microcapsules into integrated assemblies with sophisticated adaptive functionalities remains underdeveloped [27, 28]. Critical challenges include engineering systems that autonomously restrict fuel accessibility to enzymes, thereby controlling acid/base generation at target thresholds, and integrating multiple responsive capsules capable of intercommunication to dynamically govern local microenvironments [29, 30, 31]. Furthermore, precise monodispersity is essential to eliminate diffusion variability, enabling accurate spatiotemporal control of pH oscillations, thus faithfully emulating cellular uniformity and function [32, 33].

Microfluidics has emerged as a pivotal platform for generating monodisperse droplets, hydrogel particles, and for encapsulating drugs and cells [34, 35, 36, 37]. This versatility stems from microfluidic chips that enable precise flow control, in‐line manipulation, and real‐time monitoring [38, 39]. Conventional fabrication, through glass/silicon micromachining or soft lithography, requires high‐resolution molds, costly cleanroom facilities, and lengthy processing, limiting rapid iteration and commercial scalability [40, 41]. In contrast, 3D printing, particularly fused deposition modeling (FDM), enabling direct and layer‐by‐layer construction of intricate 3D geometries from digital files (e.g., CAD, SketchUp). This approach reduces time, labor, and material waste, supports integration with industrial control systems, and promotes sustainable manufacturing [42, 43, 44]. However, issues such as poor optical transparency, channel gaps leading to turbulence, wettability mismatches affecting droplet formation, and susceptibility to chemical or thermal degradation continue to challenge robust microfluidic device performance [45, 46]. Addressing these challenges is essential to fully exploit 3D‐printed microfluidic devices for monodisperse droplets formation.

**FIGURE 1:**
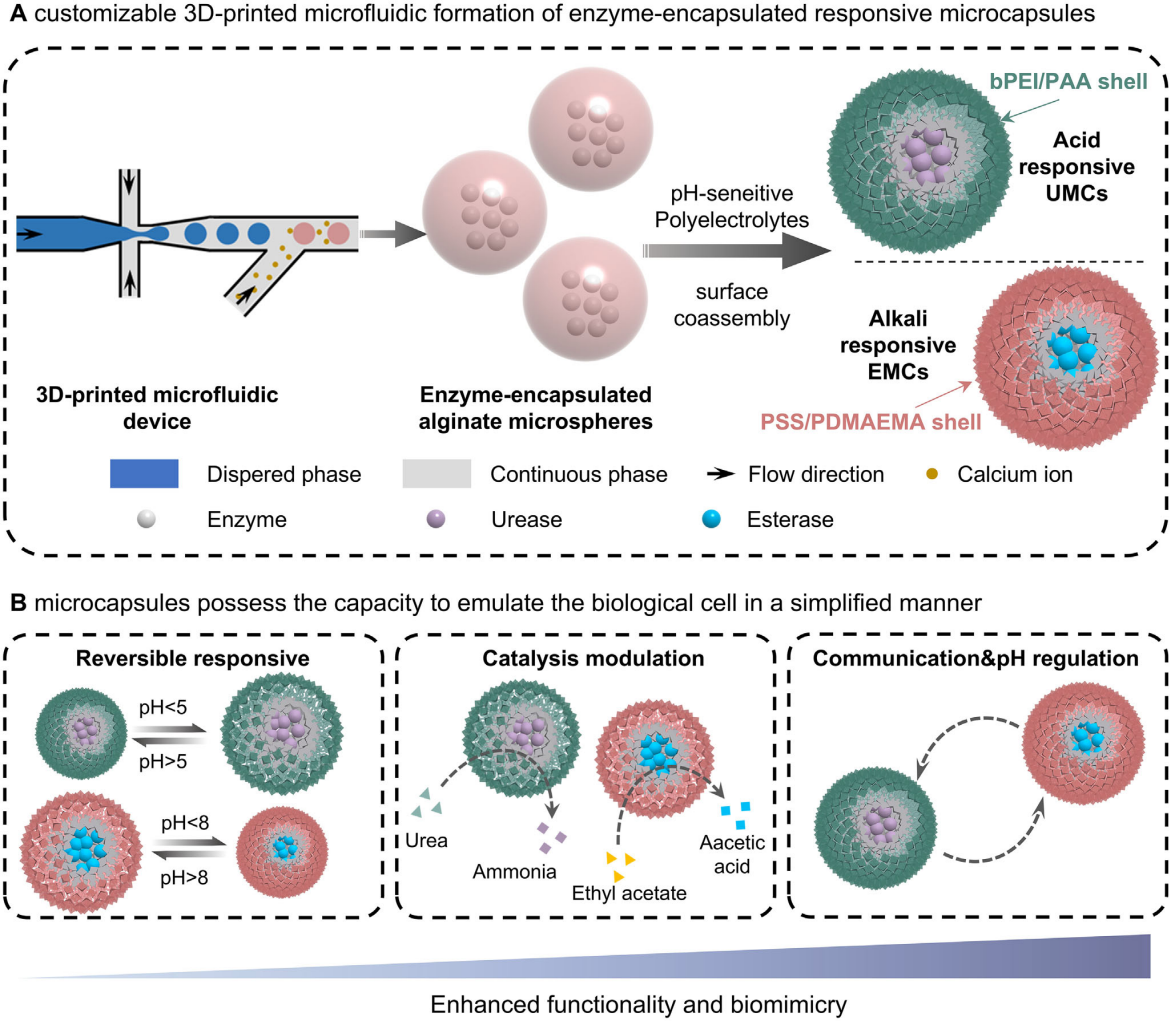
Schematic illustration for the design, fabrication, and functions of homeostatic microcapsules system. (A) The customizable 3D‐printed microfluidic formation of enzyme‐encapsulated alginate microspheres, which were subsequently deposited by polyelectrolytes to prepare responsive microcapsules through surface co‐assembly strategy. (B) The microcapsules possess the capacity to emulate the structure of a biological cell in a simplified manner, these are reversible responsive function in response to a change in environmental pH, catalysis reactions within the distinct compartments, cross‐communication, and programmable pH stabilization.

Here we report a synthetic system comprising two types of pH‐responsive microcapsules that dynamically communicate and achieve programmable pH regulation, collectively sustaining homeostatic oscillations and damped steady states of pH (Figure 1). These microcapsules were fabricated using highly customized and cost‐efficient (< $10) 3D‐printed microfluidic devices combined with surface co‐assembly strategy. Urease‐encapsulated microcapsules (UMCs) generate base, and their responsive shells are constructed from alternating layers of branched polyethylenimine (bPEI) and poly(acrylic acid) (PAA), feature selectively permeable at acidic pH < 5. Esterase‐encapsulated microcapsules (EMCs) produce acid, and their responsive shells are formed through alternate deposition of poly(sodium 4‐styrenesulfonate) (PSS) and poly[(2‐dimethylamino) ethyl methacrylate] (PDMAEMA), respond permeable at pH > 8. A novel post‐coating approach was developed to modify microfluidic channels, enabling the production of monodisperse and stable enzyme‐loaded alginate cores for subsequent responsive shell deposition. Individually, these microcapsules display precise pH‐triggered permeability, enabling controlled uptake or release of small molecules to drive enzymatic pH control. In mixed systems, UMCs and EMCs engage in pH‐mediated cross‐communication and feedback‐controlled shell dynamics, resulting in tunable homeostatic oscillations and programmable pH stabilization. The closed‐loop microcapsule system achieves bidirectional pH regulation in dispersed suspension without complicated fabrication. The system also realizes a unique dual‐functional regulation mode that integrates pH oscillations and programmable terminal pH stabilization, which could be simply adjusted by changing capsule and fuel ratios. The low‐cost devices and simple fabrication strategy also provide great potential for scalable production of monodisperse enzyme‐loaded microcapsules. This work establishes a versatile framework for constructing adaptive, life‐like systems that emulate simplified cellular regulation, opening avenues for next‐generation intelligent materials with autonomous, bioinspired functionality.

## 2 Results and Discussion

### 2.1 Design and Fabrication of FDM 3D‐Printed Microfluidic Devices

**FIGURE 2:**
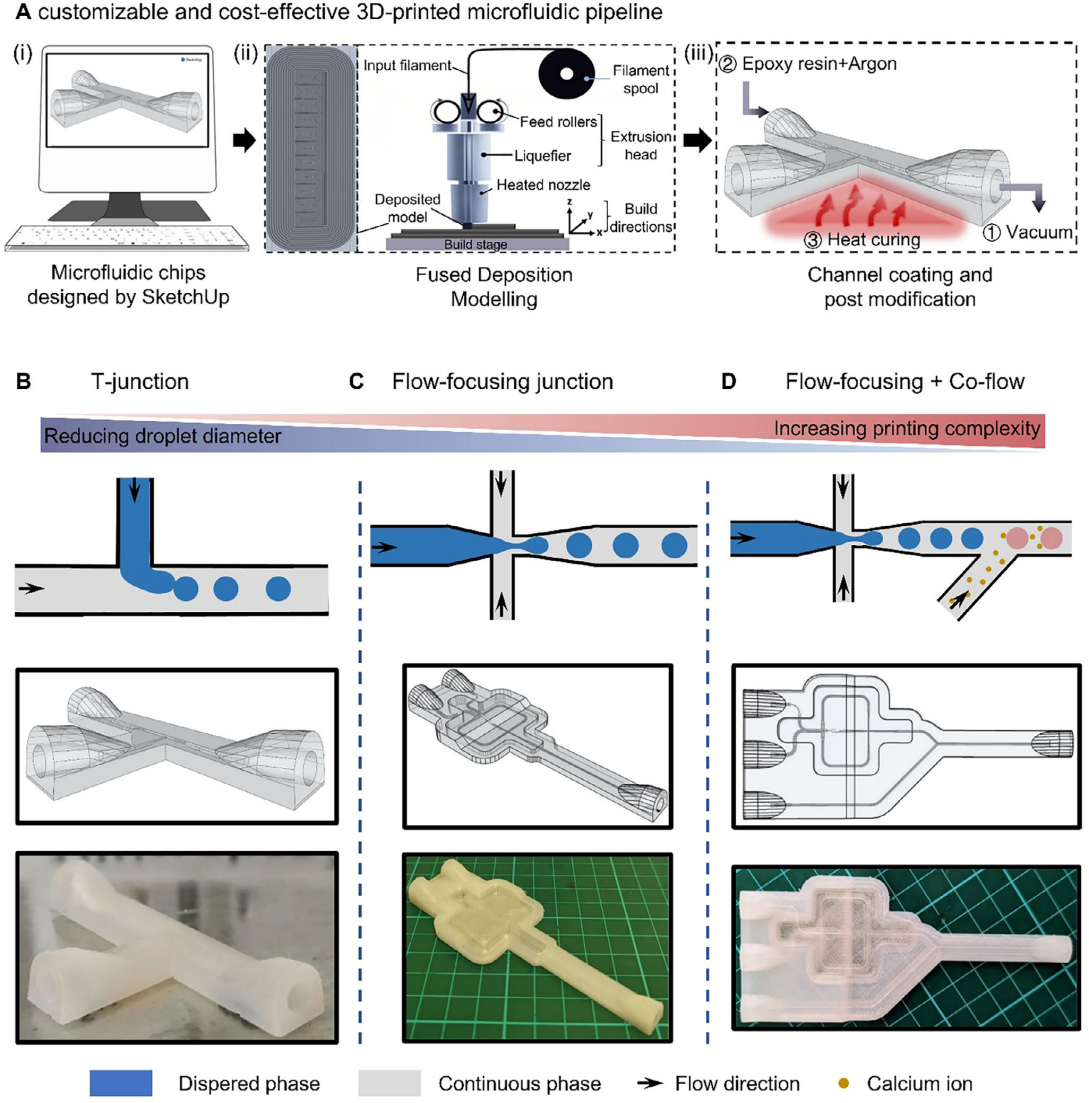
An overview of the new manufacturing pipeline of FDM 3D‐printed microfluidic chip. (A) The whole fabrication process of 3D‐printed microfluidic devices, including (i) implementing rational modeling designs using SketchUp design software, (ii) deposit fused extruded layers of Nylon filament with defined cross‐sectional area, and (iii) post‐coating treatment for microfluidic channels. (B–D) Schemes (top), designed microfluidic models (middle), fabricated microfluidic chips (bottom) for microfluidic droplet generation with different types of junctions. (B) T‐junction (10 cm length, 5 cm width), droplets formation is dominated by shear forces. (C) Flow‐focusing junction (10 cm length, 2 cm width), droplets formation is controlled by high shear and elongational stresses, which imposed by the fast‐moving continuous phase cause the dispersed thread to thin dramatically. (D) Flow‐focusing +Co‐flow hybrid junction (10 cm length, 3 cm width), droplets formation is the same as flow‐focusing regime, plus an in‐chip cross linking through a side channel injecting calcium ions.

We developed versatile microfluidic devices for generating W/O droplets and alginate microspheres using a low‐cost FDM 3D printer and an innovative, easily adaptable fabrication protocol (Figure 2A). Although FDM offers an attractive route for producing microfluidic chips, it suffers intrinsic limitations requiring careful analysis and targeted improvements. Key drawbacks include imperfect filament fusion at channel intersections, inadequate layer adhesion leading to structural weaknesses and susceptibility to compressive fractures, and limited resolution that restricts achievable microchannel dimensions. To address these challenges, we established a comprehensive workflow encompassing rational chip design via SketchUp and precise adjustment of printer parameters for high‐resolution fabrication. Additionally, we introduced a channel modification technique that enables controlled surface properties, facilitating the production of highly monodisperse, stable droplets.

This approach stands out for its exceptional customizability, adaptability, and ease of use, facilitating the printing of complex, precise microfluidic structures with minimal effort. Printer configuration and device design require integrated decisions on filament type, port geometry, base layout, and related architectural features (Figures S1–S3). A critical aspect of device design is the junction geometry, which directly controls droplet generation. We systematically investigated T‐junctions, flow‐focusing junctions, and a novel hybrid (flow‐focusing + co‐flow) configuration. In T‐junctions, the dispersed phase meets the continuous phase perpendicularly, relying on shear forces at the corner to break the stream into droplets (Figure 2B). Flow‐focusing junctions introduce dual continuous‐phase channels that converge, imposing strong shear and elongational stresses that thin the dispersed stream into droplets (Figure 2C). However, we found that during off‐chip crosslinking of alginate droplets, instabilities led to irregular microspheres, compromising subsequent microcapsule fabrication. To address this, a flow‐focusing and co‐flow hybrid microfluidic (FCMF) was designed, featuring an in‐chip pre‐crosslinking channel where calcium ions are introduced, stabilizing alginate droplets in situ (Figure 2D). This ensures the formation of monodisperse, robust alginate microspheres in the future bath crosslinking.

### 2.2 Post Coating Modification and Optimization of Printing Parameters

**FIGURE 3:**
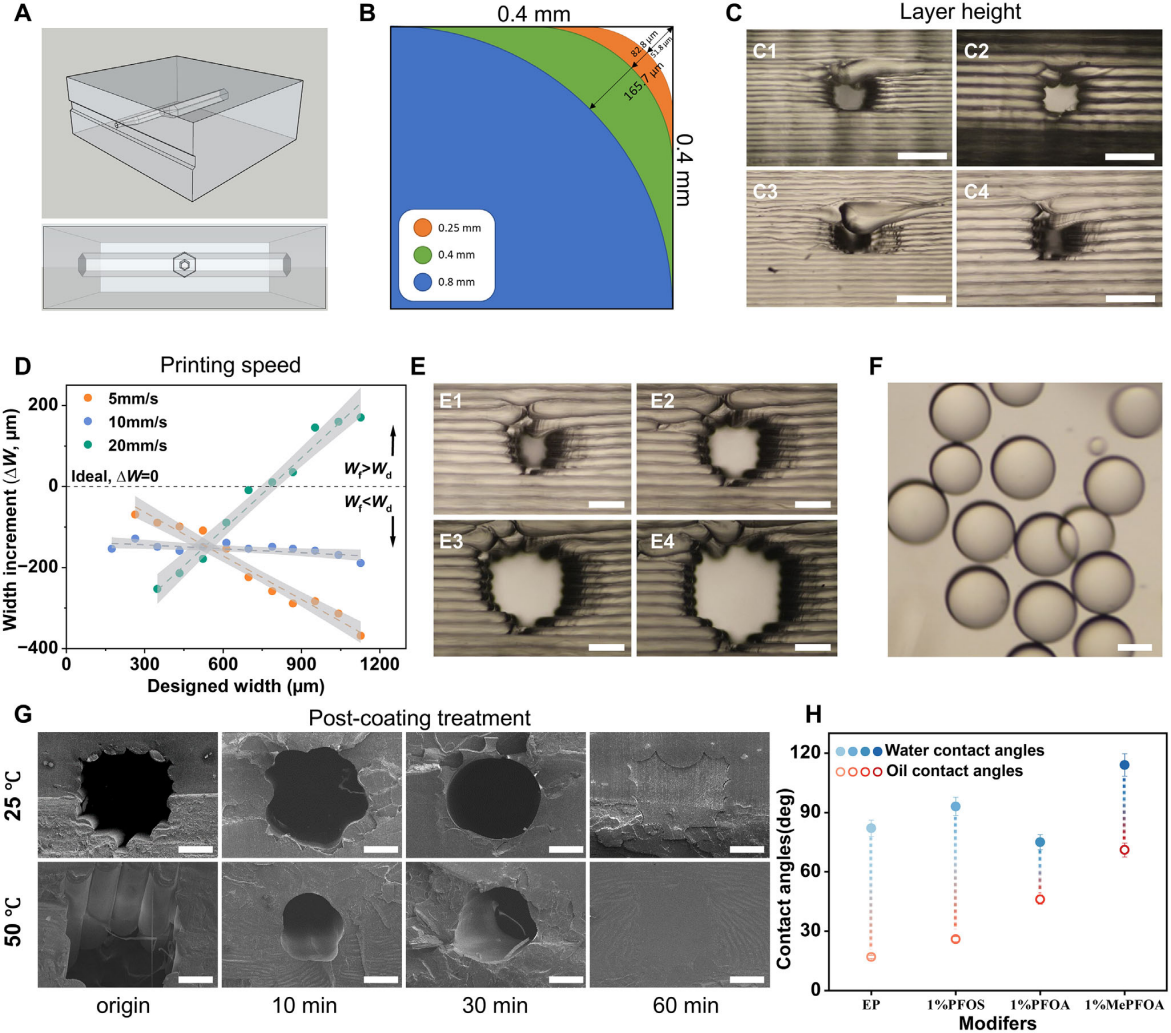
Optimized strategy for fabricating 3D‐printed microfluidic devices with prescribed channel geometries and post coating modification. (A) The three‐stage design of dispersed phase channel with a 265 µm width channel (400 µm long) before widening to the 435 µm width channel, then to 790 µm width channel. The continuous phase channel has a 790 µm width channel. (B) Illustration of how different nozzle widths determine corner sharpness. (C) Channels printed with different layer height and nozzle diameters. The layer height and nozzle diameters are (C1) 0.05, 0.25; (C2) 0.10, 0.25; (C3) 0.05, 0.40; (C4) 0.10, 0.40 mm. (D) Fabricated channel width at different printing speeds of 5, 10, and 20 mm/s. (E) The difference between fabricated and designed width, with examples at (E1) 435, (E2) 615, (E3) 790, and (E4) 955 µm, respectively. (F) Droplets formed through 265 µm width dispersed‐phase and 790 µm width continuous‐phase channels. (G) Images of channels before and after allowing the epoxy resin coating to cure for 10, 30, and 60 min at room temperature and 50°C. (H) Addition of different hydrophobic/lipophobic modifiers to epoxy resin to affect the contact angle of water and oil. Experiments were performed in triplicate, and results are presented with standard deviations to reflect reproducibility and measurement precision.

To successfully fabricate the designed FCMF microfluidic chip, it is essential to optimize FDM printing parameters to mitigate inherent deviations between digital models and printed outputs, particularly for channel geometries critical to forming monodisperse alginate microspheres. Evaluation commenced with the three‐stage dispersed‐phase channel, a critical determinant of droplet formation dynamics (Figure 3A, Figures S4 and S5). Adjustable parameters include channel and junction geometry, printhead, printing speed, and layer height. Direct comparisons of designed vs. printed cross sections revealed characteristic FDM distortions, necessitating informed design compensations. For instance, hexagonal channels with a height increased by ∼15% (100%/cos30°) counteract ceiling droop, achieving improved dimensional fidelity (Figure S6). Printhead selection also significantly impacts outcomes. Nozzle diameters (0.25, 0.4, 0.8 mm) directly influence resolution (Figure S7). Due to resolution constraints, 0.8 mm was excluded. Figure 3B demonstrates that larger nozzles exacerbate corner rounding. In terms of the microfluidic device, if any angles are printed sharper than 135°, then it will go beyond the printer's capabilities, and the different nozzles will be an important factor in junction design. The appropriate combination of nozzle diameter and layer height is critical, as these parameters must be jointly tuned to maximize printing resolution and structural integrity. Figure 3C reveals that halving the layer height to 0.05 mm compromises print integrity. At this resolution, the printer operates near its mechanical limits, leading to notable defects, particularly fluctuations in layers beneath the channel (Figure 3C1,C3). Although the lower channel surface appears smoother, the upper channel exhibits significant over‐extrusion, where excess filament accumulates at corners, potentially impairing droplet formation. Consequently, 0.05 mm layers were excluded due to print irregularities and prolonged fabrication times, though it may suit channels < 100 µm if technical challenges are resolved. Also, the designed model was rigorously assessed during G‐code generation to ensure accurate translation of design features and to optimize channel capacity (Figure S8). Collectively, the combination of a 0.10 mm layer height and 0.25 mm nozzle achieved the best compromise between dimensional properties and structural robustness, establishing a solid foundation for droplet generation performance.

Using these optimized parameters, we further investigated the effect of printing speed on channel performance (Figure 3D). Although higher speeds (20 mm/s) reduce print time, they often compromise quality. Printing at slower speeds (5 mm/s) improves mechanical robustness yet significantly increases build time and yields unpredictable channel dimensions, particularly for larger channels. Prolonged molten filament residence likely promotes over‐fusion, thereby disrupting fidelity to the designed channel geometry. Channels were printed to assess width variations with odd and even layers on separate designs, starting hexagonal features after 16 layers (Tables S1 and S2). The results revealed that channels were on average 185 µm narrower and junctions were 24 µm wider than designed (Figure 3E and Figure S9). This optimization led to an ideal combination: AA printhead, 0.25 mm nozzle, 0.10 mm layer height, and 10 mm/s speed. FCMFs fabricated under these conditions successfully generated water‐in‐oil droplets (Figure 3F). Although most droplets maintained a uniform diameter of approximately 300 µm, occasional smaller droplets were observed, attributed to inner wall roughness and micro‐defects that disrupted laminar flow and induced localized turbulence, ultimately affecting droplet uniformity.

To mitigate the mentioned intrinsic FDM limitations, such as internal gaps inducing turbulence, dispersed‐phase adhesion compromising droplet formation, and vulnerability to chemical or thermal degradation, we developed a post‐coating treatment strategy using modified epoxy resin. This method effectively repairs defects, smooths internal surfaces, and imparts amphiphobic properties with minimal geometric distortion. Resin deposition and residence time are finely tuned to produce smooth wall coatings. Excess epoxy is cleared by laminar flow, maintaining an unobstructed central conduit for fluid transport. Cross‐sectional scanning electron microscope (SEM) images provide a clear visualization of the coating formation procedures inside the channels (Figure 3G). Uncoated channels showed substantial roughness from incomplete layer alignment, which likely promotes turbulence, undermining droplet stability. Coating‐treated channels exhibited progressive wall coverage. After 10 min of curing time, at both temperatures (25°C and 50°C), coatings smoothed gaps significantly but remained conformed to them, showing an irregular circle. At 30 min, the coating established a near‐circular cross‐section, overriding gap‐induced irregularities, denoting optimal curing. Beyond 60 min, excessive viscosity prevented effective channel clearing. With 30 min, temperature optimization indicated that curing at 50°C produced indistinct coating‐device interfaces, implying superior adhesion relative to room temperature, enhancing the longevity and mechanical resilience of the modified channels.

The omniphobic behaviors of coating with different fluorinated modifiers were systematically investigated. The epoxy coating with 1% MePFOA exhibits exceptional omniphobic behavior toward liquids, demonstrating a water contact angle of 118° ± 1.5° and an oil contact angle of 70° ± 1.7° (Figure 3H). Notably, MePFOA imparted substantial amphiphobicity even at 0.1%, elevating water contact angles from 82° to 109° and dramatically increasing cyclohexane and octane contact angles from 17° and 13° to 68° and 63°, respectively (Figure S10). This mainly originates from synergistic surface effects that include low surface free energy and enhanced surface mobility facilitated by multiple –CF_2–_ groups in the modifier. Collectively, this strategy establishes a robust framework for fabricating FDM‐based microfluidic devices that produce droplets exhibiting superior stability and monodispersity compared to previously reported systems.

**FIGURE 4:**
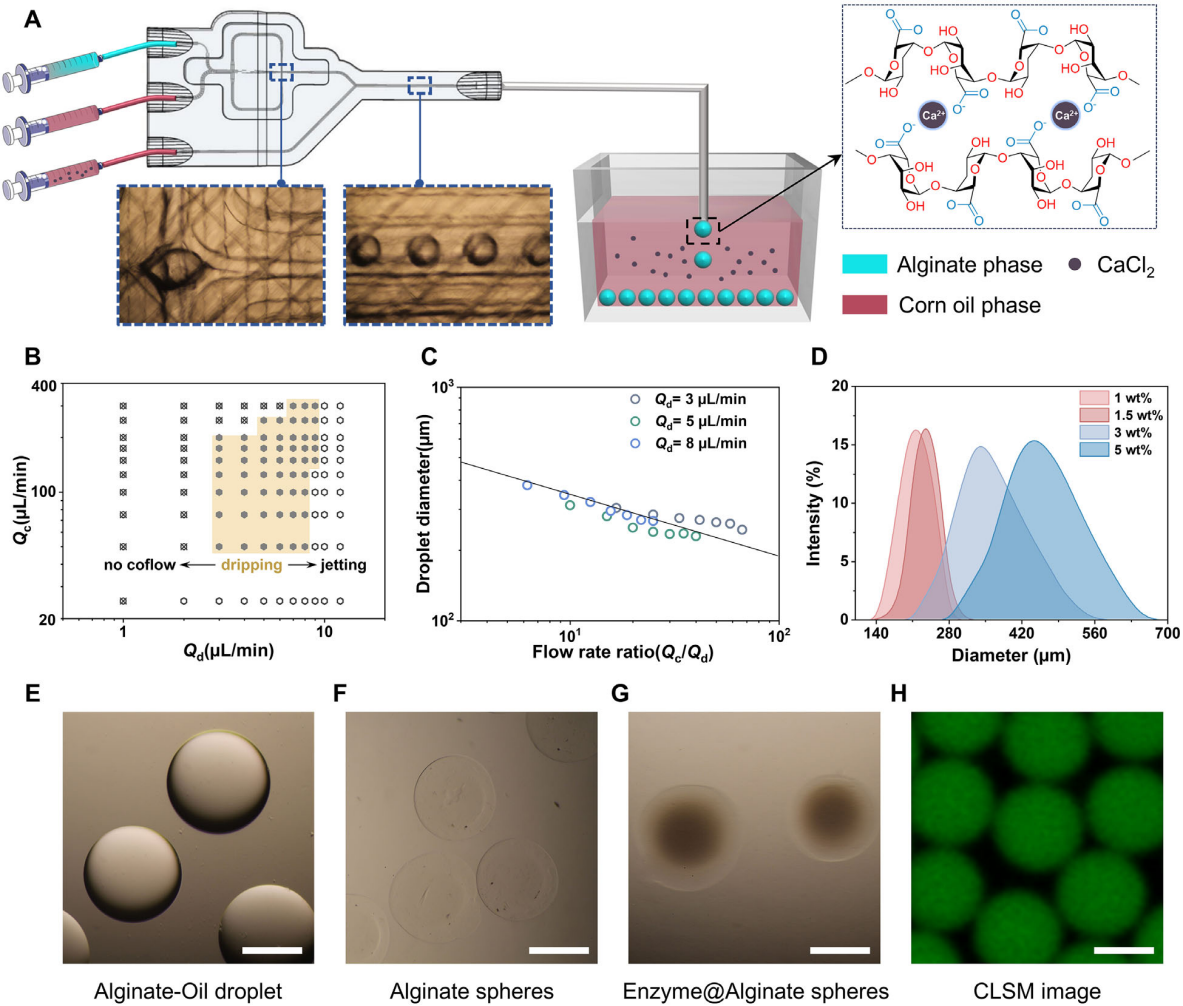
FCMF enables controlled production of spherical alginate microspheres. (A) Schematic illustration of the microfluidic setup. The alginate is cross‐linked by Ca^2+^ ions to form stable microspheres. (B) Phase diagram spanned by (*Q*_c_, *Q*_d_) with indicated flow patterns observed in a microfluidic device. (C) Plots showing the effect of the flow rate ratio (*Q*_c_/*Q*_d_) on droplet diameter. (D) The variation of spherical alginate beads diameter with different Na‐alginate concentrations. FCMF operated in dripping mode enabled the production of monodisperse (E) alginate in oil emulsion droplets, (F) alginate microspheres, (G) enzyme‐encapsulated alginate microspheres, (H) CLSM image of enzyme‐encapsulated alginate microspheres. Scale bars, 200 µm.

The experimental setup employs an FCMF device featuring three inlets (for dispersed alginate, continuous oil, and CaCl_2_ crosslinking stream) and an outlet discharging into an open container for secondary crosslinking (Figure 4A). Dispersed alginate and continuous oil phases were infused via syringe pumps with optimized flow rates, ensuring alginate is sheared into W/O droplets at the junction. The droplets were then partially pre‐crosslinked by CaCl_2_ from the third inlet, and subsequently discharged into the container for complete crosslinking, yielding robust and stable microspheres (Figure 4A inset). At constant channel dimensions, the flow rate ratio of dispersed (*Q*_d_) to continuous phases (*Q*_c_) critically governs droplet monodispersity and stability. To validate size tunability, Q_d_ (1–10 µL/min) and *Q*_c_ (30–300 µL/min) were systematically adjusted, revealing diverse flow regimes and corresponding droplet diameters (Figure 4B). These results confirm the device's capacity to precisely modulate droplet formation across a broad range of flow conditions. Flow regimes depend critically on the relative velocities of the dispersed and continuous phases. At high dispersed‐phase and low continuous‐phase velocities, the phases flow in parallel, forming a dispersed jet (jetting mode). When flow rates are suitably optimized, the dispersed phase breaks into droplets carried by the continuous phase, achieving the dripping mode. Alternatively, rapid continuous‐phase flow coupled with slow dispersed flow traps the dispersed phase within its inlet channel, precluding interfacial flow. Uniform channel dimensions maintain laminar regimes, enhancing reproducibility (Movies S1 and S2). Under dripping conditions, droplet generation achieves high monodispersity, with diameter coefficients of variation (CV) typically ranging from 1% to 3%. Here, the CV [47] is defined by the ratio of the standard deviation (*δ*) to the average value (*d*_av_) of the droplet diameter shown below in Equation (1).

(1) $$\mathrm{CV}\;=\frac{\delta }{d_{av}}\;\times 100\%$$

To enhance the universality and reproducibility of our FCMF platform, we supplemented the capillary number (Ca) and Weber number (We), the core dimensionless numbers governing microfluidic droplet generation, to quantify our operating regime. For our stable dripping system, where droplet breakup is dominated by the shear force of the continuous oil phase, Ca and We are defined as:

(2) $$C_{a}=\frac{\mu_{c}\upsilon_{c}}{\gamma }\;$$

(3) $$W_{e}=\frac{\rho_{c}\upsilon_{c}^{2}D_{h}}{\gamma }\;$$

Under the optimized conditions for monodisperse microsphere fabrication (Q_d_ = 5 µL/min, *Q*_c_ = 100 µL/min), we obtained Ca = 5.56 × 10^−3^ and We = 5.89 × 10^−4^, which fall within the stable dripping window. The detailed calculation formulas, fluid parameters, and results are provided in Tables S1 and S2.

Several flow velocities within the optimal range were explored to analyze how the flow rate ratio of the continuous to dispersed phase (*Q*_c_/*Q*_d_) influences droplet size (Figure 4C). As *Q*_c_/*Q*_d_ increased from 6.3 to 66.7, droplet diameters decreased from 400 to 225 µm, achieving greater stability than the T‐junction configuration (Figures S11, S12, and Movie S5). Additionally, due to the pronounced viscosity of alginate aqueous phase, its concentration variations notably affect droplet dimensions by modulating shear and extensional forces at the junction. For hydrogel encapsulation, 2–4 wt.% alginate is generally optimal to support enzyme viability and proliferation [48]. Viscosity analyses performed at room temperature on 1–5 wt.% alginate solutions demonstrated a strong positive correlation between droplet size and alginate concentration, attributed to viscosity effects (Figure 4D and Figure S13). In the dripping regime, monodisperse alginate droplets were achieved at lower concentrations (1 or 1.5 wt.%), whereas higher concentrations (3 or 5 wt.%) resulted in larger, more polydisperse droplets. Optimal conditions were identified using 1.5 wt.% alginate at 5 µL/min and a continuous phase at 100 µL/min, yielding monodisperse alginate‐in‐oil droplets of ∼235 µm (Figure 4E, Figure S14, Movies S3, S4, and S6). Subsequent pre‐gelation from the third channel yielded robust crosslinked and enzyme‐encapsulated microspheres (Figure 4F,G and Figure S15). Confocal laser scanning microscope (CLSM) images revealed homogeneous enzyme distribution, indicative of effective encapsulation for biocatalysis (Figure 4H and Figure S16). Collectively, these results highlight the FCMF platform's efficacy in fabricating stable, monodisperse alginate emulsions and microspheres.

### 2.3 Responsive Microcapsules Preparation and pH Regulation Behaviors

**FIGURE 5:**
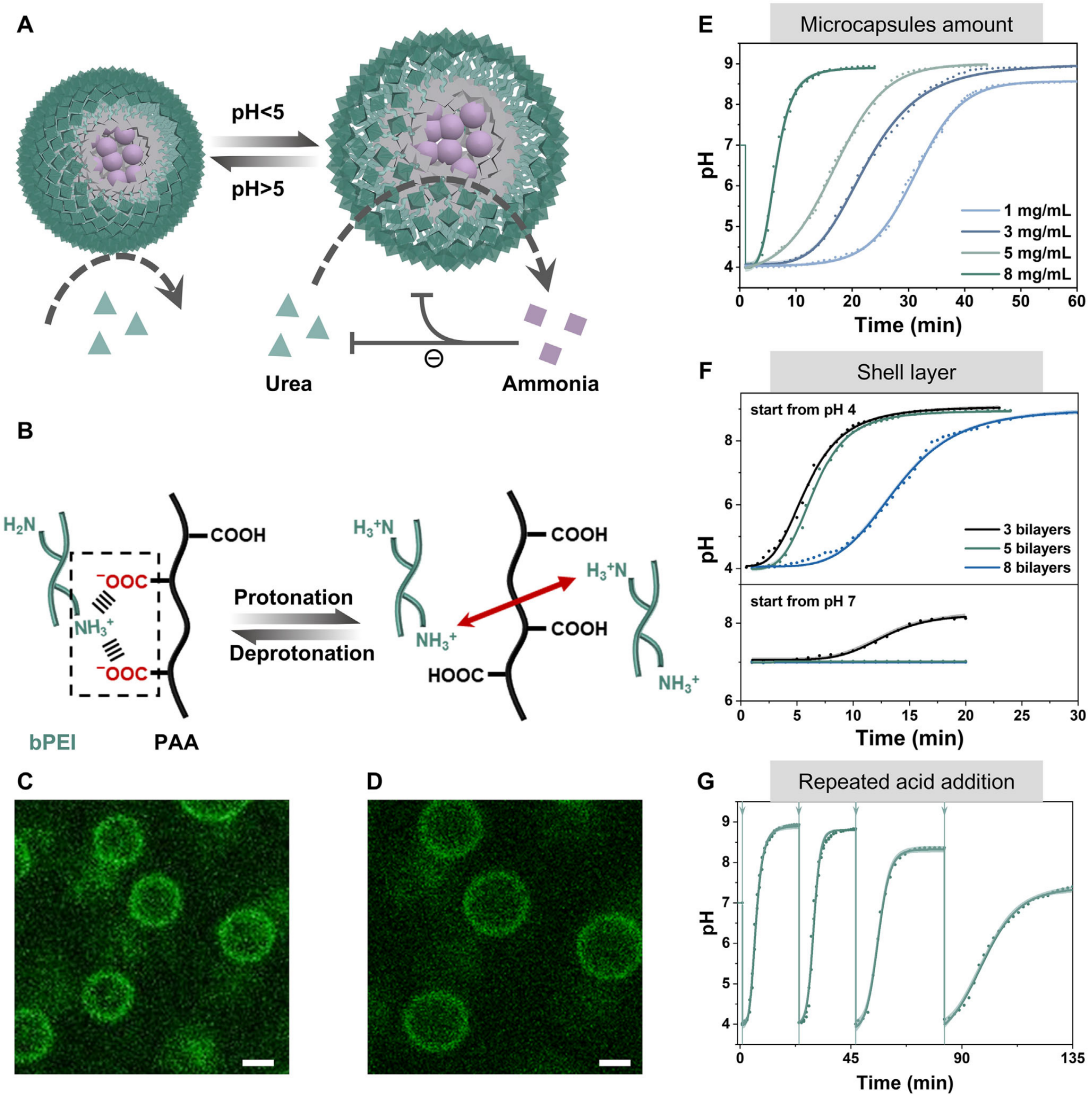
Acid‐mediated automodulation of UMCs and base‐generation regulation behavior. (A) Schematic representation of acid‐responsive biocatalytic hydrolysis of urea to produce ammonia by UMCs. The production of accumulated ammonia, generating a positive feedback for pH increasing and a negative feedback for microcapsule permeability. (B) The working mechanism of acid‐responsive reversible permeability modulation of shell of UMCs. (C) CLSM images of UMCs at pH 7, the shell is impermeable, close state. (D) CLSM images of UMCs at pH 4, the shell is permeable. Effect parameters on pH modulation for UMCs. (E) Variation of microcapsules amount. Urea concentration: 100 mm. (F) Effect of shell layers thickness. Microcapsule concentration: 5 mg/mL; Urea concentration: 100 mm. (G) The repetitive acid stimuli lead to repetitive pH self‐regulation behavior. In each cycle, the dispersions pH was adjusted to 4 using 0.25 m HCl solution, stabling for 1 min and monitoring the pH changes. Microcapsule concentration: 5 mg/mL; Urea concentration: 100 mm. Shell layers: 5 bilayers. All data are an average of three measurements (*n* = 3 technical replicates, mean ± s.d.). Scale bars, 200 µm (unless otherwise indicated).

Acid‐responsive urease microcapsules (UMCs) were fabricated by coating monodisperse enzyme‐loaded alginate microspheres with polyelectrolytes via a layer‐by‐layer co‐assembly strategy (Figure 5A). The responsive shell, constructed from alternating layers of branched polyethylenimine (bPEI) and poly(acrylic acid) (PAA), exhibits reversible permeability based on pH‐triggered protonation/deprotonation of functional groups (Figure 5B). The PAA has a well‐defined pKa of 4.5–5.0 from carboxylic acid groups. Below pH 5, PAA's carboxyl groups become protonated, while bPEI amines generate electrostatic repulsion, promoting structural swelling and enhancing permeability. This facilitates the diffusion of urea to initiate the biocatalytic reaction by encapsulated urease. Ammonia generated by enzymatic catalysis elevates the local pH, exerting a positive regulatory effect on the microenvironment. This pH shift simultaneously induces a negative feedback response by decreasing the permeability of the pH‐responsive shell, thereby modulating substrate diffusion and suppressing further enzymatic activity. CLSM imaging of UMCs (containing outermost FTIC‐labeled bPEI) confirms pH‐induced diameter transitions, from 343 µm (pH 7, impermeable “closed” state) to 448 µm (pH 4, permeable “open” state) (Figure 5C,D and Figure S17).

To investigate pH‐mediated feedback and self‐regulating behaviors, parameters such as microcapsule amount, shell layer number, urease, and urea concentrations were systematically explored (Figure 5E–G and Figure S18). For UMCs (5 mg/mL urease encapsulated), the increasing UMC concentration significantly accelerated the pH elevation rate. The time required for a pH shift from 4 to 8.7 was reduced from 53 to 17 min as the UMC concentration increased from 1 to 8 mg/mL (Figure 5E). As a deciding factor, the pH‐responsive functionality of the UMC shells with varying bPEI/PAA bilayer numbers (each bilayer formed via one deposition cycle) was assessed for their permeability regulation behaviors under different pH conditions (Figure 5F). Shells with 5 or 8 bilayers exhibited effective pH‐gated permeability, remaining impermeable at pH 7 to restrict urea diffusion, but switching to a permeable state at pH 4 to enable substrate uptake and initiate enzymatic reactions. This validates the role of the shell in both urea absorption and alkali product diffusion through a feedback‐regulated mechanism by pH change. Notably, at pH 4–5, diffusion‐driven processes of urea and the alkali products dominate pH elevation. In contrast, the 3‐bilayer shell exhibited leakage at pH 7, confirming inadequate barrier integrity in the “close” state. Thus, precise control of bilayer number enables pH‐gated transitions between “open” and “close” states of UMCs. Furthermore, within practical ranges, increasing urease (>5 mg/mL) or urea (>100 mm) concentrations did not significantly impact ammonia generation (Figure S18). Repetitive pH regulation was demonstrated using UMCs (5 mg/mL urease) with an initial urea fuel (100 mm). HCl was introduced periodically to reset pH to 4 after each plateau, achieving multiple cycles (Figure 5G). The final cycle terminated at pH ∼7, because the system has run out of the background fuel urea. Collectively, these results confirm the pH‐driven negative feedback regulation characteristic of UMCs (Figure S21A), wherein elevated pH decreases shell permeability, thus modulating catalytic activity, enabling self‐regulated pH homeostasis within the microenvironment.

**FIGURE 6:**
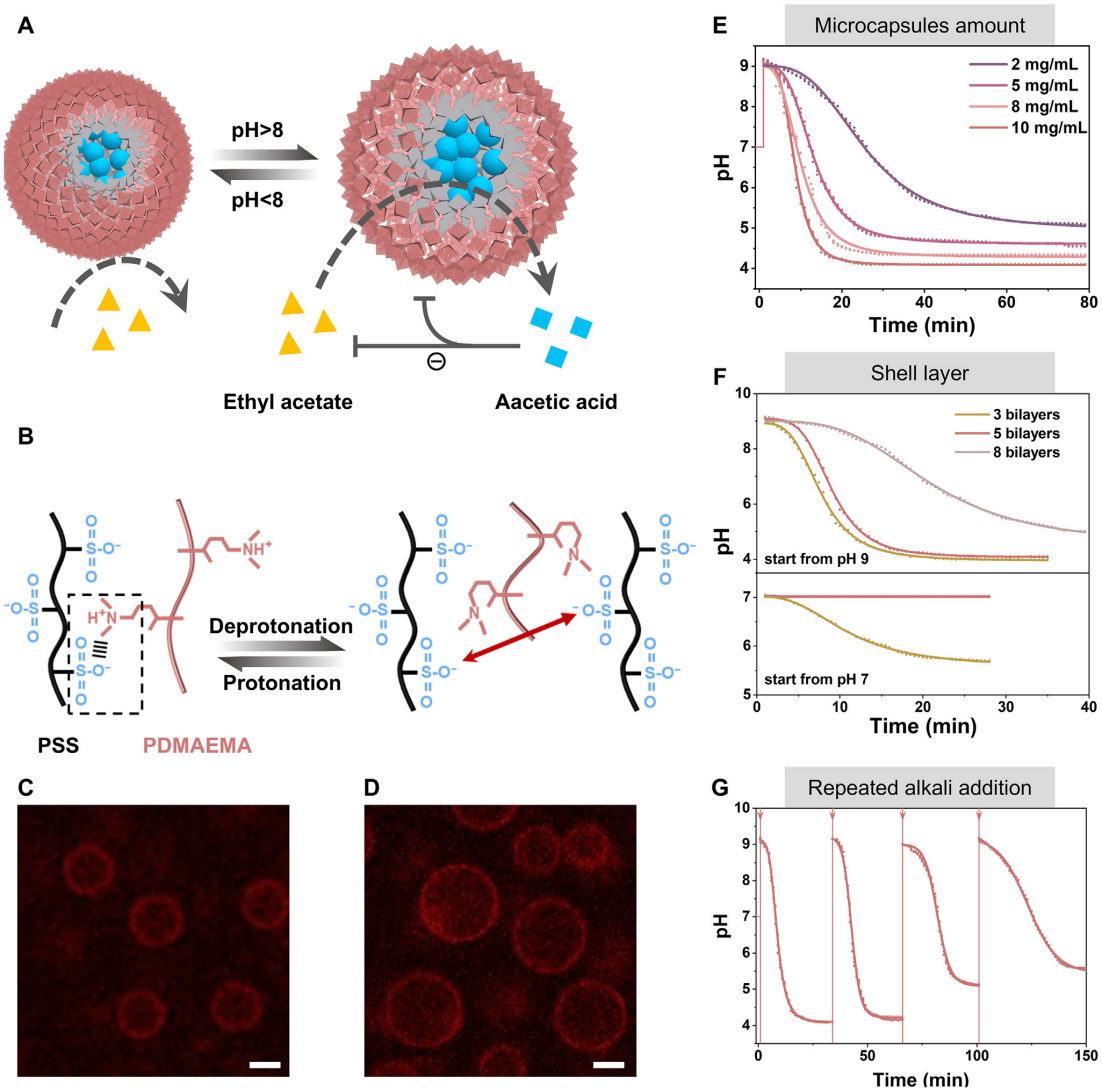
Alkali‐mediated self‐modulation of EMCs and acid‐generation regulation behavior. (A) Schematic representation of alkali‐responsive biocatalytic hydrolysis of ethyl acetate to produce acetic acid by EMCs. The production of accumulated acid, generating a positive feedback for pH decreasing and a negative feedback for microcapsule permeability. (B) The working mechanism of alkali‐responsive reversible permeability modulation of shell of EMCs. (C) CLSM images of EMCs at pH 7, the shell is impermeable, close state. (D) CLSM images of EMCs at pH 9, the shell is permeable, open state. Effect parameters on pH modulation for EMCs (E) Variation of microcapsules amount. EA concentration: 100 mm. (F) Effect of shell layers thickness. Microcapsule concentration: 8 mg/mL; ethyl acetate (EA) concentration: 100 mm. (G) The repetitive alkali stimuli lead to repetitive pH self‐regulation behavior. In each cycle, the dispersions pH were adjusted to 9 using a 0.25 m NaOH solution, stabling for 1 min and monitoring the pH changes. Microcapsule concentration: 8 mg/mL; Urea concentration: 100 mm; Shell layers: 5 bilayers. All data are averages of three measurements (*n* = 3 technical replicates, mean ± s.d.). Scale bars, 200 µm (unless otherwise indicated).

Adjusting pH to an alkaline state alone does not fulfill the criteria for adaptive homeostatic regulation. Thus, we explored how to enable autonomous pH regulation oscillating between acidic and basic states, eventually reaching a damped steady state. Hence, alkali‐responsive esterase microcapsules (EMCs) were constructed via a polyelectrolyte co‐assembly strategy on esterase‐loaded alginate microspheres. The EMC shell was formed through alternate electrostatic deposition of poly(sodium 4‐styrenesulfonate) (PSS) and poly[(2‐dimethylamino) ethyl methacrylate] (PDMAEMA), exhibiting an “open” state at pH > 8 (Figure 6A). As PSS is a strong polyelectrolyte, it is hardly affected by pH change, shell responsiveness primarily depends on the pH‐sensitive PDMAEMA. The PDMAEMA is a weak cationic polyelectrolyte with a pKa of 7.5–8.0. Upon protonation, the amine functionalities of PDMAEMA undergo pH‐dependent conformational transitions (Figure 6B). A crumple and cyclic structure predominates at pH > 8, while a linear conformation is favored at pH < 7. The crumpled structure increases the porosity within the shell layer structure. Combined with the electrostatic repulsion of the sulfonic acid groups in PSS, these structural features collectively promote shell swelling and increased permeability under alkaline conditions. CLSM analysis confirms the pH‐dependent permeability. EMCs remain impermeable at pH 7 diameter of around 352 µm, while at pH 8 they switch to a swollen and permeable state with a diameter of around 511 µm (Figure 6C,D and Figure S19).

The systematic evaluation of key parameters, including microcapsule amount, shell layers, urease concentration, urea concentration, reveals their effects on pH regulation dynamics (Figure S20). The concentration of EMCs is positively correlated with the rate of pH regulation and the time it takes to reach the final equilibrium value (Figure 6E). Similar to the UMC system, higher enzyme or substrate concentrations accelerated the pH shift, although this effect plateaued beyond 8 mg/mL esterase and 100 mm ethyl acetate (EA). Owing to esterase's comparatively lower activity, higher enzyme and substrate levels were required to achieve similar regulation kinetics. Even under optimized conditions, EMCs took longer to reach pH equilibration. Additionally, the three‐layered EMC shell failed to fully restrict EA diffusion at pH 7, while 5‐bilayer structures offered optimal control (Figure 6F). Upon repeated alkali additions, the pH decayed over time in a similar fashion to the first cycle, until a plateau value was again reached (Figure 6G). This system, therefore, represented a second pH‐mediated feedback controlled loop in which the acidity increased (due to the production of acetic acid) to deactivate further catalysis by EMCs (Figure S21B). Given that the pH regulation activity of EMCs led to a transition of the environmental pH (from pH ∼9 to pH∼4), these results highlight the potential to reinitiate the catalytic activity of UMCs when mixing both microcapsules populations. The static and dynamic enzyme leakage after microcapsule preparation and pH oscillation experiments were also evaluated via UV–vis spectrophotometry. The low enzyme leakage rate of the microcapsule system is a key prerequisite for the realization of inter‐capsule communication. The negligible free enzymes in the bulk solution ensure that the pH oscillation is completely driven by the localized signal exchange between the two types of microcapsules, which further enhances the potential of inter‐capsule chemical communication (Figures S22 and S23).

### 2.4 Homeostatic pH Regulation in Dual‐Microcapsule System

In many organisms, cell‐to‐cell communication is a fundamental mechanism for constructing intercellar networks and maintain quorum response on the external impacts [49]. We sought to replicate these natural responses by developing a self‐regulating microcapsules system capable of generating homeostatic pH oscillations and programmed pH stabilization via an antagonistic enzymatic reaction network.

**FIGURE 7:**
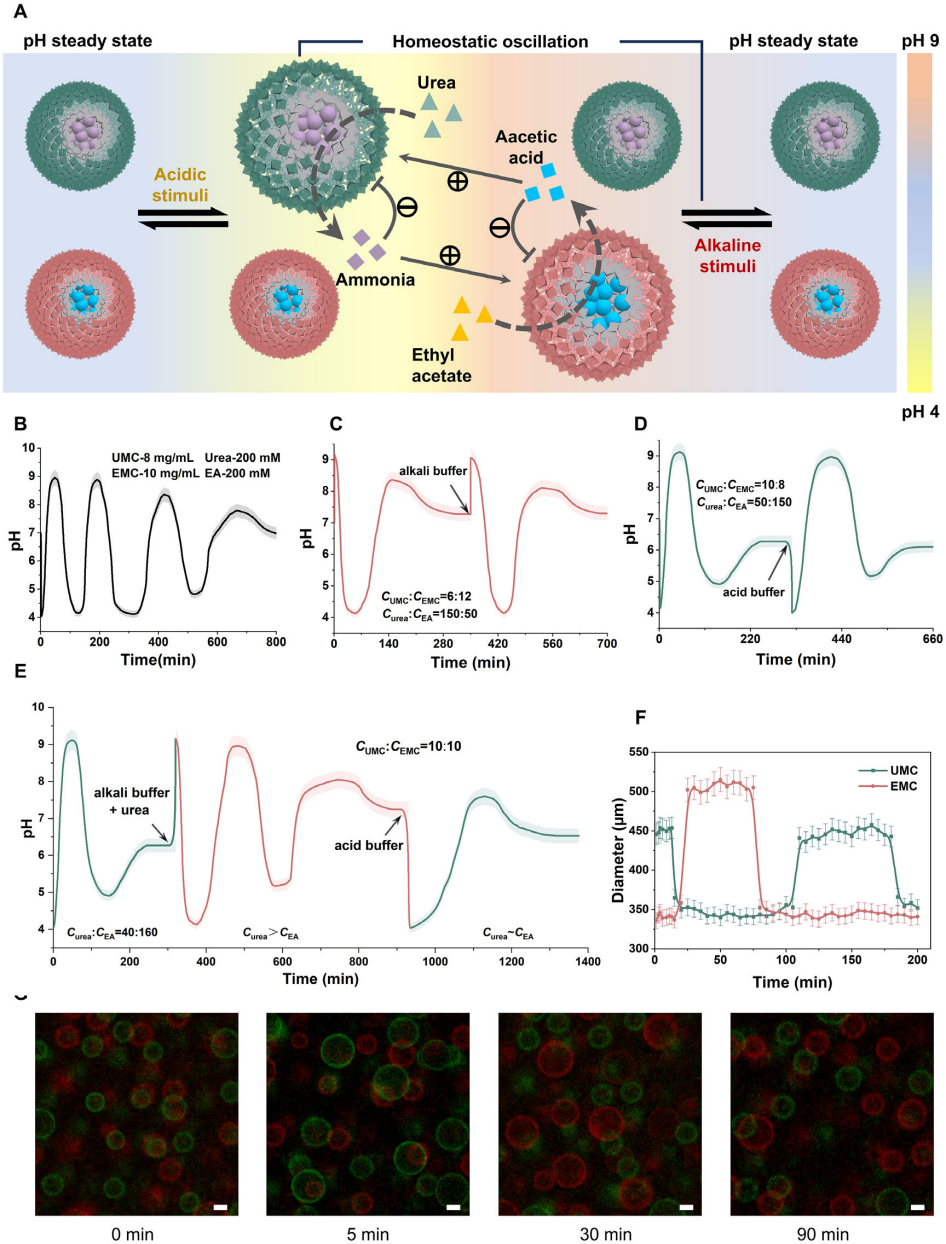
Collective pH auto‐modulation behaviors by inter communication in microcapsule spheroids and feedback‐controlled programmable pH stabilization. (A) Schematic representation of non‐equilibrium homeostatic oscillation by modulation of pH by UMCs and EMCs in the presence of acidic or alkali stimuli. From left to right: acidic stimuli, which UMC start first; from right to left: alkali stimuli, which EMC start first. (B) The pH oscillations when the mixed microcapsule system was triggered by an acidic stimuli. Conditions: 8 mg/mL UMC, 10 mg/mL EMC, 200 mm urea, and 200 mm EA. (C) Programmed pH stabilization at around 7 when the mixed microcapsule system was triggered by alkali stimuli. Conditions: *C*_UMC_ + *C*_EMC_ = 18 mg/mL, with a ratio of 6:12; *C*_urea_ + *C*_EA_ = 200 mm, with a ratio of 150:50. (D) Programmed pH stabilization at around 6 when the mixed microcapsule system was triggered by acidic stimuli. Conditions: *C*_UMC_ + *C*_EMC_ = 18 mg/mL, with a ratio of 10:8; *C*_urea_ + *C*_EA_ = 200 mm, with a ratio of 50:150. (E) Robust pH stabilization behaviors between pH 6 and 7 when the system was stressed by external acidic or alkali variation. Conditions: *C*_UMC_ + *C*_EMC_ = 20 mg/mL, with a ratio of 10:10; *C*_urea_ + *C*_EA_ ∼ 200 mm during the whole process, with three different ratios from starting to ending. (F) The diameter variations of UMCs and EMCs of the first 200 min during the pH regulation process in (E). (G) Merged CLSM images of UMCs and EMCs at selected time points, which come from the first 90 min during the pH regulation process in (E). (*n* = 3 technical replicates, mean ± s.d.). Scale bars, 200 µm (unless otherwise indicated)

To induce nonequilibrium pH homeostatic oscillations, UMCs and EMCs were co‐integrated into a single aqueous system containing urea and ethyl acetate (EA) as fuels (Figure 7A). The system was initially stable at neutral pH, subsequently was activated by an acidic stimulus, which reduced the pH from ∼7 to 4 (Figure 7B). This triggered the UMCs switched to “open” state with a higher permeability while EMCs remained impermeable, which led to urease‐catalyzed urea hydrolysis to produce ammonia. As expected, the pH increased and accumulated ammonia provides a negative feedback to UMC's permeability, reached a transition plateau at pH 8.8 over 45 min. Upon reaching the transition pH (∼8.8), the accumulated ammonia enhanced EMC permeability through a positive feedback loop, as EMCs become permeable at pH > 8. This pH‐responsive shift activated esterase within EMCs, which then assumed regulatory dominance as UMCs entered a dormant state. The resulting acidification gradually reduced the pH to ∼4.2 over the next 85 min. This cyclic activation‐deactivation of opposing microcapsules, governed by pH‐responsive permeability, enabled self‐sustained oscillations until substrate depletion drove the system to a damped steady state. With successive oscillation cycles, the amplitude of pH fluctuations diminished, as reflected by a declining alkaline plateau and a rising acidic plateau. Upon alkaline stimulation, the out‐of‐equilibrium dynamics were reinitiated, with EMCs responding first.

We further investigated pH regulation behaviors under varying microcapsule ratios and external stimuli (Figure S24). Different UMC/EMC ratios produced distinct pH fluctuation patterns and final pH stabilization values (Figure S25). Notably, both UMCs and EMCs retained their structural integrity and functional responsiveness throughout repeated pH cycling, demonstrating the robustness and precision of this feedback‐governed homeostatic regulation under nonequilibrium conditions. The observed regulatory dynamics suggest an alternative application. Through precise modulation of capsule composition and fuel input, the system enables programmable pH control, including predefined oscillation count and terminal pH stabilization. This adaptability positions the system as a versatile platform for tailored microenvironmental regulation in diverse biochemical or physiological contexts.

Unlike prior cases exhibiting repeated large‐amplitude pH oscillations culminating in a damped equilibrium, the system allows for precise stabilization at a target pH value by predefining the microcapsule composition and fuel input. The feasibility of this programmed pH homeostatic control under alkaline conditions was experimentally confirmed (Figure 7C). To achieve a pre‐programmed pH steady state, a system incorporating a UMC/EMC ratio of 6:12 and a urea/EA ratio of 150:50 (totaling 200 mm) was employed. Upon exposure to an alkaline stimulus, EMCs activated first, lowering the pH to ∼4.1. This in turn triggered UMC activity, raising the pH to ∼8.3. Due to the limited working window (pH > 8) for EMC activity, the subsequent acidification resulted in a damped stabilization at pH ∼7. The system reached this preprogrammed stable pH in approximately 280 min, which is significantly faster and more accurate than multi‐cycle oscillatory systems (∼800 min). The relatively low UMC content only allowed pH recovery to ∼8.3, just sufficient to activate EMCs. Likewise, limited EA content restricted acid production, preventing further pH drops, thus achieving the purpose of pre‐programmed pH value regulation in this system. This precise regulation arises from a dormant pH window (5 < pH < 8), where neither capsule is active. At this range, EMC‐generated acid imposes negative feedback to themselves without reinitiating the positive feedback for UMC catalysis, enabling accurate control over the final pH. Thus, by modulating fuel and capsule ratios, the activation/dormant periods can be strategically programmed via feedback‐controlled mechanism to achieve defined pH steady‐state behaviors. We further investigated pre‐programmed pH regulation under acidic stimulation (Figure 7D). Using a UMC‐to‐EMC ratio of 10:8 and a urea‐to‐EA ratio of 50:150 (total 200 mm), the system achieved a damped stabilization pH of ∼6.3. This stabilization was also governed by a feedback‐controlled mechanism, wherein the second activation window of UMCs enabled localized ammonia production, which imposed negative feedback on UMC permeability while remaining below the threshold required to initiate EMC activation, thus halting further catalytic cycling.

To further validate the system's capacity to regulate pH under complex environmental perturbations, we conducted a long‐duration test using alternating acid–base stimuli (Figure 7E). The system, formulated with UMC:EMC at 10:10 and an initial urea:EA at 40:160 (totaling ∼200 mm), was exposed to a sequential three‐stage “acid‐alkali‐acid” stimuli. Remarkably, the system exhibited stable homeostatic regulation behaviors, maintaining pH values between 6 and 7. Simultaneous morphological analysis revealed dynamic size fluctuations of individual microcapsules, consistent with their permeability transitions, yet the capsule architecture remained structural integrity throughout the reversible expansion–contraction cycles (Figure 7F). CLSM imaging further confirmed that the functional integrity of individual microcapsules was preserved within the integrated system, supporting their stable and prolonged operation in maintaining pH homeostasis (Figure 7G). Also, we further performed single‐factor control simulations to distinguish the contributions of substrate depletion, enzyme activity decay, and shell diffusion kinetics to the observed regulation behavior. The results showed that substrate depletion and enzyme activity decay mainly change the damping rate of oscillation amplitude, making a negligible contribution to the pH oscillation behavior. The shell response kinetics is the key determinant of pH regulation behavior, governing the features of the initial oscillatory pattern, including regulation cycle, amplitude, and period (Figures S27–S30). Overall, pH homeostasis in the microcapsules system is achieved through intercommunication and feedback‐regulated interactions between two distinct microcapsule populations, UMCs and EMCs. This engineered platform conceptually emulates the adaptive microenvironmental regulation observed in multicellular assemblies, while its stability and programmability underscore its potential in the development of next‐generation intelligent materials.

## 3 Conclusion

In this work, we present a bioinspired microcapsule system that autonomously modulates pH through enzymatic feedback, similar to homeostatic regulation in nature. By integrating a low‐cost FDM 3D‐printed microfluidic platform with a rationally optimized fabrication workflow, we overcame intrinsic limitations of FDM printing through targeted design strategies, precision printing parameters, and an epoxy‐based post‐coating treatment. The resulting hybrid flow‐focusing/co‐flow junction and defect‐free, amphiphobic channels are achieved for the precision fabrication of monodisperse enzyme‐loaded alginate microcapsules. Our system comprises urease‐ and esterase‐loaded microcapsules with pH‐responsive shells: UMCs activate in acidic conditions to release base, while EMCs activate in alkaline conditions to release acid. Individually, each microcapsule type demonstrated delayed, pH‐triggered permeability, enabling temporally controlled molecular exchange. In mixed populations, they communicate via pH‐mediated feedback, generating antagonistic enzymatic loops that sustain oscillations or achieve programmable stabilization. This communication network remains robust under repeated acid/base perturbations, reflecting the resilience of natural homeostatic systems.

Despite the robust pH homeostasis performance of dual‐microcapsule system, its long‐term operation is intrinsically fuel‐limited. The pH regulation behaviors will terminate upon the depletion of substrates, which may challenge this system in practical applications. The modular design of our system provides universality to address such limitations. The regulation mechanism relies on the antagonistic enzymatic pH‐regulation network, which is controlled by a designed pH‐triggered shell. This makes the replacement of enzymatic reaction modules and relevant fuel substrates to match the target applications is possible, without changing the regulation mechanism of the dual‐microcapsule system. For example, the system can use endogenously abundant substrates (e.g., circulating urea, physiological lipids, or esters) as fuel to achieve self‐regulation without exogenous supplementation in vivo biomedical applications. For environmental regulation, the system can be integrated with slow‐release carriers encapsulated substrates to extend its operation time.

Our system demonstrates a broadly applicable design paradigm for constructing adaptive, self‐regulating materials that couple microfluidic precision, responsive polymer architectures, and programmed reaction networks. While exemplified here in pH homeostasis, this strategy can be extended to create autonomous, signal‐communicating materials for complex tasks in biosensing, pollutant neutralization, and precision therapeutic platforms.

## Author Contributions

Conceptualization: H.D.Z., J.S., D.S. Methodology: H.D.Z., J.S., D.S. Investigation: H.D.Z., J.S., R.C., D.S. Visualization: H.D.Z., J.S., R.C., D.S. Validation: H.D.Z., J.S., R.C., D.S. Formal analysis: H.D.Z., J.S., R.C., D.S. Supervision: H.Y.W., D.S. Writing – original draft: H.D.Z., R.C., R.U. Writing – review & editing: H.D.Z., H.Y.W., D.S. Project administration: H.D.Z., D.S. Funding acquisition: D.S.

## Funding

This work was financially supported by the EPSRC projects EP/Y030397/1 and EP/X039773/1.

## Conflicts of Interest

The authors declare no conflicts of interest.

## Supporting information




**Supporting File 1**: smll73756‐sup‐0001‐SuppMat.docx.

**Supporting File 2**: smll73756‐sup‐0002‐MovieS1.mp4.

**Supporting File 3**: smll73756‐sup‐0003‐MovieS2.mp4.

**Supporting File 4**: smll73756‐sup‐0004‐MovieS3.mp4.

**Supporting File 5**: smll73756‐sup‐0005‐MovieS4.mp4.

**Supporting File 6**: smll73756‐sup‐0006‐MovieS5.mp4.

**Supporting File 7**: smll73756‐sup‐0007‐MovieS6.mp4.

## Data Availability

The data that support the findings of this study are available from the corresponding author upon reasonable request.
