# Supplementary material for: Autonomous and Communicative Microcapsule Systems for Life‐Like Homeostatic pH Regulation
Source: Small. 2026 May 13;22(37):e73756. doi: 10.1002/smll.73756 (PMC13325715; doi:10.1002/smll.73756)
Supplement: Supplementary file 1 — Supporting File 1: smll73756‐sup‐0001‐SuppMat.docx. [file SMLL-22-e73756-s007.docx]

Supporting Information

Autonomous and Communicative Microcapsule Systems for Life-Like Homeostatic pH Regulation

Hongda Zhou, James Smith, Rui Cheng, Ramzan Ullah, Huaiyuan Wang, Dmitry Shchukin*

*Corresponding author. Dmitry Shchukin Email: d.shchukin@liverpool.ac.uk

**This PDF file includes:**

Materials and methods

Figs. S1 to S30

Tables. S1 to S6

Legends for movies S1 to S6

**Other Supplementary Materials for this manuscript include the following:**

Movies S1 to S6

**Materials and Methods**

**Materials**

Alginic acid sodium salt from brown algae (SA), corn oil, calcium chloride, urease from Canavalia ensiformis (Jack bean, Type III, powder, 15,000–50,000 units/g solid), esterase from porcine liver (lyophilized powder, ≥15 units/mg solid), urea (99.0%, solid), ethyl acetate (EA, anhydrous, 99.8%), bovine serum albumin (BSA, lyophilized powder, ≥95%), branched polyethylenimine (bPEI, average Mw ∼ 25000 g/mol), poly(acrylic acid) (PAA, average Mw ∼ 450000 g/mol), poly(sodium 4-styrenesulfonate) (PSS, average Mw ~70,000), poly[(2-dimethylamino) ethyl methacrylate] (PDMAEMA, average Mw ∼ 10000 g/mol), Rhodamine B isothiocyanate (RBITC), Fluorescein isothicyanate (FITC), citric acid (CA), sodium citrate (Na3C), octane, cyclohexane and anhydrous ethanol were purchased from Sigma-Aldrich, U.K. and used without further purification. Polypropylene (PP), poly lactic acid (PLA) and Nylon were supplied by UltiMaker (Netherlands). The Milli-Q water was used throughout all experiment procedures.

**Fabrication of 3D-printed microfluidic devices**

*The structure design of microfluidic devices*

The microfluidic devices modelling were designed using a SketchUp software and exported as obj. files. Key structural components included the base, fluidic channels, and inlet/outlet ports. The base was designed to resist deformation and fracture under operational loads. Channel widths (265–1045 µm) were selected to ensure printability, minimize hydraulic resistance, and allow reliable droplet formation and collection. Various filament materials, including PP, PLA, and nylon, were evaluated for compatibility and structural performance. Two droplet generation junctions, T-junction and flow-focusing, were designed and adapted depending on droplet size, channel length, and the fittings used. Fig. S3 shows representative base and channel configurations, while Fig. S2 illustrates the outlet port design. The overall design, especially channel alignment and junction configuration, must ensure smooth delivery of both phases and unobstructed droplet release. While user-driven design flexibility is retained, minimalism and reliability are prioritized.

*3D printing of microfluidic devices*

Microfluidic devices were printed using fused deposition modeling (FDM). The designed models (obj. files) were converted into G-code using Cura software, applying slicing parameters optimized for the selected 3D printer (UltiMaker, Netherlands). Among the tested filaments, nylon was selected for its superior optical clarity, mechanical strength, and resistance to organic solvents, making it most suitable for microfluidic applications. Multiple device configurations were evaluated. The finalized design used in subsequent experiments includes three inlets for the inner aqueous phase, continuous oil phase, and outer oil phase, and a single outlet for droplet collection. Devices were printed layer-by-layer, following standard FDM procedures. Prior to printing, nylon filaments were dried under vacuum at 50 °C overnight. Key printing parameters included: nozzle diameter 0.25 mm, layer height 0.1 mm, print speed 10 mm/s, extrusion temperature 220 °C, and bed temperature 85 °C.

**Channel coating post-treatment**

To minimize turbulent flow and enhance droplet stability in W/O emulsions, a post-fabrication surface modification was developed to render microfluidic channels amphiphobic. A modified epoxy resin was selectively deposited to coat the channel walls without altering overall morphology or blocking flow. Leveraging laminar flow, the resin adhered only to the inner walls, leaving a central conduit for fluid passage. The process began by activating the channel surface. Devices were cleaned with 10% ethanol under vacuum to remove debris, followed by water rinsing and vacuum drying. A resin mixture (30 μL/cm channel length) was introduced via the attachment port and guided through the channel using argon flow. After 10 minutes of static orientation, additional argon pressure created a central tunnel by displacing unbound resin. The device was then cured at room temperature or 50 °C for 10–60 minutes to determine optimal conditions, followed by nitrogen flushing (10 mL/s) to remove excess resin. This coating process was repeated to further refine the cross-sectional area. Finally, channels were rinsed repeatedly with acetone and ethanol.

**Formation of enzyme-encapsulated alginate microspheres**

*The W/O emulsions formation and pre-gelation*

All microfluidic experiments were conducted using a pressure-driven pump system (Pump 11 Elite Series, Harvard Apparatus, USA) and 9 mL stainless steel syringes (Luer-Lok, Harvard Apparatus). An optical microscope equipped with a Canon EOS 1100D digital SLR camera (12 MP APS-C sensor) was used for image acquisition. The microfluidic device was assembled using injection syringes, capillary tubes, needles, and PVC tubing. Water-in-oil (W/O) alginate emulsions were generated via a flow-focusing junction. These droplets subsequently passed through a co-flow junction, where they underwent pre-gelation upon contact with an outer oil phase containing dispersed CaCl₂. The inner aqueous phase, consisting of SA, BSA, and enzymes (urease or esterase), was injected at a rate of 5 μL/min. The continuous oil phase was corn oil, while the outer oil phase comprised corn oil with suspended CaCl₂ particles, delivered at 100 μL/min.

*The collection and formation of alginate microspheres*

The pre-gelation alginate microspheres were collected in glass vessels (e.g., vials or Petri dishes) containing the outer oil phase and incubated for 2 hours to ensure complete ionic crosslinking. Due to their higher density relative to the oil, the microspheres sedimented and were subsequently retrieved from the bottom using a custom-made mesh holder composed of a glass support (KG-25, ADVANTEC, Japan) and a 42 μm nylon mesh (Tokyo Screen, Japan). Residual CaCl₂ emulsion and corn oil were removed via sequential washing with hexane and deionized water (three cycles each). The purified, enzyme-encapsulated alginate microspheres were then dispersed in water in a Petri dish for subsequent experiments.

**Preparation of acid-responsive urease microcapsules (UMCs)**

The inner aqueous phase was freshly formulated by combining SA, BSA, and urease in a mass ratio of 20:5:1. The outer oil phase was prepared by sonicating CaCl₂ powder into corn oil to achieve a 1.5 wt% suspension. Urease-loaded alginate microspheres were subsequently generated via the microfluidic method described above.

For interfacial layer-by-layer (LBL) polyelectrolyte assembly, bPEI and PAA were dissolved separately in 500 mL of deionized water to prepare 2 wt% and 1 wt% solutions, respectively, with the pH adjusted to ~6.5. A volume of 25 mL of bPEI solution was then added to urease-encapsulated microspheres solutions (1 g/mL, 25 mL). The mixture was gently agitated for 30 minutes at ambient temperature to facilitate electrostatic adsorption of the positively charged bPEI onto the particle surfaces. After filtration and washing with deionized water to remove non-adsorbed bPEI, the microspheres were treated with 25 mL of PAA solution under identical conditions to form a negatively charged outer layer. Repetition of this process yielded multilayer-coated urease microcapsules (UMCs). This entire process was described as a complete LBL surface coassembly process, the resulting acid-responsive UMCs were subsequently washed, filtered, and air-dried at room temperature.

**Preparation of alkali-responsive esterase microcapsules (EMCs)**

The esterase-encapsulated alginate microspheres were produced using the same microfluidic platform as described previously. The inner aqueous phase was freshly prepared by dissolving SA, BSA and esterase in a weight ratio of 20:8:1. For LBL surface coassembly process, 1.5 wt% solutions of PDMAEMA and PSS were prepared in deionized water with the pH adjusted to ~6.5. These polyelectrolytes were alternately deposited onto the microspheres via electrostatic assembly, forming multilayered esterase microcapsules (EMCs). The final alkali-responsive EMCs were purified by filtration, washed thoroughly with water, and dried at ambient conditions.

**Synthesis of FTIC-labeled bPEI**

To obtain FTIC-labeled bPEI, bPEI was first dissolved in sodium carbonate buffer (2 wt%, 20 mL, pH ~9) to facilitate amine deprotonation. FTIC (0.66 g) was dissolved in DMSO (4 mL) and added to the bPEI solution under continuous stirring. The reaction proceeded overnight at ambient temperature, after which the mixture was dialyzed (MWCO 3.5 kDa) against deionized water for 72 h in the dark. The purified FTIC-bPEI conjugate was immobilized onto microspheres using the same procedure employed for unmodified bPEI.

**Synthesis of RBITC-labeled PDMAEMA**

A solution of PDMAEMA in DMF (1 mL) was prepared, and RBITC (0.8 mg, 0.0014 mmol) with pyridine (8 μL, 0.10 mmol) was dissolved in another 1 mL of DMF. The solutions were combined and stirred at ambient temperature for 2 h under dark conditions. The RBITC-functionalized PDMAEMA was isolated by methanol precipitation and dried under vacuum.

**Enzyme activity and stability**

*Evaluation of encapsulated enzymes and free enzymes activity*

Catalytic activities of urease and esterase were examined across a series of pH conditions, adjusted with HCl or NaOH. Enzymes were incubated for 5 min in pH-adjusted solutions before fuel addition. Urease activity was quantified via ammonium detection using the indophenol blue method,^[1]^ whereas esterase activity was measured by UV–vis detection of acetic acid formation with phenol red as an indicator*.*^[2]^ Real-time pH changes were recorded at ambient temperature with a 4-channel pH station (EA Instruments) calibrated against standard buffers (pH 4, 7, and 10) before each experiment. All pH measurements are reported as the mean ± SD of ≥3 independent replicates.

*Evaluation of enzymatic stability of UMCs and EMCs*

Encapsulated and free enzymes were stored in phosphate buffer (pH~7) for two weeks to assess long-term stability. For UMCs and free urease, pH was adjusted to 4.0 using urea and acidic buffer (9 mM CA/Na₃C), and relative activity was determined via ammonium quantification. For EMCs and free esterase, pH was adjusted to 9.0 using EA and alkali buffer (9 mM Tris), and relative activity was determined via acetic acid quantification. All measurements are reported as the mean ± SD of ≥3 independent replicates.

*Static enzyme leakage test after microcapsule preparation*

Experimental design: Freshly prepared UMCs and EMCs were washed 3 times with PBS buffer (pH=7.4) to remove residual unencapsulated free enzymes. The microcapsules were resuspended in PBS and incubated at 25°C for 24 h. After incubation, the microcapsules were removed by centrifugation (8000 rpm, 6 min) and collected. The enzyme activity in the supernatant was quantified using UV-Vis spectrophotometry method. All experiments were performed in 3 independent replicates.

*Dynamic enzyme leakage test during pH oscillation experiments*

Experimental design: The UMCs and EMCs microcapsules oscillation experiments were set up according to the conditions in the main text. We collected the supernatant of the microcapsule systems at the end of pH oscillation experiment, and quantified the enzyme activity in the supernatant using the same UV-Vis method. All experiments were performed in 3 independent replicates.

**The reversible responsiveness and stability of microcapsules**

All microcapsule types were evaluated for reversible responsiveness via pH-induced swelling–shrinking behavior. Microcapsules (1 wt%) were dispersed in phosphate buffer (pH~7, 20 mL), and pH was adjusted between 4 and 9 using 0.25 M HCl or NaOH. After 30 min incubation, diameters were determined by widefield microscopy and DLS. Reversible cycling stability was assessed by monitoring morphological changes via CLSM over 30 swelling–shrinking cycles. Diameters were quantified using ImageJ, with at least three independent measurements for statistical reliability.

**Swelling ratio analysis**

The diameter of microcapsules at pH=7 was measured and recorded as the initial diameter (Do). Subsequently, the UMCs and EMCs (1 wt%) were dispersed in buffer solution (20 mL) at pH=4 and at pH=9, respectively. After an incubation period of 30 minutes, the diameters of UMCs and EMCs were measured and recorded as the terminal diameter (Dt) again. The swelling ratio was calculated using the formula as follow.

$$Swelling ratio=100\%*\left( Dt-Do \right)/Do$$

**Optical morphology and size analysis**

To assess emulsion formation in the newly developed compact 3D-printed device, the morphologies of alginate emulsions and microspheres were examined using an optical microscope (Canon EOS 1100D DSLR, 12 MP APS-C sensor). Size measurements for 50 individual emulsions or microspheres were obtained via ImageJ, and statistical plots were visualized using Origin.

**Contact angle analysis**

For the epoxy resin modifications, the hydrophobicity and lipophobicity were measured via the DSA100 Expert Drop Shape Analyser (Kruss Scientific, Germany). The automated system with 5 µL droplets permitted reproducible dynamic contact angles.

**CLSM Image acquisition and processing**

To visualize cyclic “swelling-shrinking” dynamics, FITC-labeled bPEI and RBITC-labeled PDMAEMA was used to stain the microcapsule. Confocal laser scanning microscopy (CLSM, Leica SP8, tunable white light laser) enabled two-channel imaging at 492 and 561 nm excitation, respectively. Image datasets were processed and quantified using ImageJ.

**pH regulation measurements**

To elucidate the feedback mechanisms in the urea–urease and EA–esterase reaction networks, the pH-regulation behaviors of UMCs and EMCs were examined under systematically varied conditions, including microcapsule concentration, number of deposited layers, enzyme loading, and fuel concentration. For UMCs, suspensions containing 1–8 mg/mL microcapsules in phosphate buffer (pH~7, 10 mL) were acidified to pH 4 with 1 M HCl, stabilized for 1 min, and triggered by adding urea solutions (50–200 mM). For EMCs, 2–10 mg/mL microcapsules in buffer (pH~7) were alkalinized to pH 9 with 1 M NaOH, stabilized for 1 min, and initiated with EA solutions (50–200 mM). Cyclic pH-regulation experiments employed identical protocols, with pH reset between cycles using 1 M HCl or NaOH. pH profiles were recorded in real time on a four-channel pH station (EA Instruments) at room temperature, with electrodes calibrated (pH 4, 7, 10) before each run. All pH measurements are reported as the mean ± SD of ≥3 independent replicates.

**Evaluation of base-induced negative feedback-loop of UMCs by addition of free urease**

To verify that the cessation of urease catalysis was not due to fuel exhaustion but mediated by pH-triggered shell permeability, we conducted experiments probing the negative feedback mechanism in UMCs. Following the acid-stimulated pH regulation protocol (see Methods), upon reaching a steady-state pH plateau (~7.5), 1 mL of 1 wt% free urease solution was introduced. The subsequent pH increase was monitored over time, confirming that enzymatic activity is modulated by shell permeability changes rather than substrate limitation.

**Evaluation of acid-mediated negative feedback-loop of EMCs by addition of free esterase**

Analogous experiments were conducted to confirm that the acid-induced negative feedback on esterase activity in EMCs results from pH-dependent modulation of shell permeability. Base-triggered pH regulation assays were performed as detailed in the Methods. Upon reaching a steady-state pH plateau (~6), 1 mL of 1 wt% free esterase solution was introduced, and the ensuing pH decrease was monitored over time, validating acid-mediated feedback regulation via shell permeability.

**Communication and programmed pH stabilization within microcapsules system**

We investigated inter-microcapsule communication and programmed pH stabilization by integrating urease-encapsulated microcapsules (UMCs; 5 mg/mL urease, 5 bilayer shells) and esterase-encapsulated microcapsules (EMCs; 8 mg/mL esterase, 5 bilayer shells) within a single system. The total microcapsule concentration was maintained at 18 mg/mL, with a combined fuel concentration of approximately 200 mM. Dynamic communication and pH modulation was externally triggered via 1 M HCl or NaOH, initiating catalytic feedback between the two capsule populations. Systematic variation of microcapsule and fuel ratios enabled tuning of steady-state pH values. For neutral pH stabilization (~7), UMC:EMC and urea:EA ratios of 6:12 and 150:50 were employed, respectively. The system was initially alkalinized to pH 9 and subjected to repeated alkali additions to assess cyclic stability. Acidic stabilization (~6) was achieved using UMC:EMC and urea:EA ratios of 10:8 and 50:150, respectively, with initial acidification to pH 4 and repeated acid stimuli challenges. Mixed pH stabilization was evaluated under alternating acid-base stimuli with UMC:EMC and urea:EA ratios of 10:10 and 40:160. Morphological changes and diameter fluctuations of FITC- and RBITC-labeled microcapsules were captured by CLSM, with dual-channel images merged and quantified using ImageJ, demonstrating coordinated microcapsule communication underpinning programmable pH homeostasis. All pH measurements are reported as the mean ± SD of ≥3 independent replicates.

**Dual chamber experiment**

The dual-chamber experiment was performed using a home-made acrylic dual-chamber device with a single chamber volume of 25 mL and an effective membrane contact area of 2.0 cm2. A regenerated cellulose dialysis membrane with a molecular weight cutoff (MWCO) of 3500 Da was used to separate the two chambers, which was pre-treated by soaking in deionized water for 30 min to remove residual preservatives, and rinsing three times with fresh deionized water before use. UMCs and EMCs used in this experiment were same as the experiments in the main text, with consistent particle size, enzyme loading, and pH-responsive performance.

The left chamber was filled with 25 mL of UMC suspension containing 100 mM urea, the right chamber was filled with 25 mL of EMC suspension containing 100 mM ethyl acetate, both chambers were initially adjusted to pH 7.0. The experiment was initiated by acidifying the left chamber to pH 4.0 with acidic buffer to activate UMCs. All experiments were performed at 25 °C, the pH of each chamber was continuously monitored in real time using pH meters, and all tests were performed with 3 independent replicates.

Supplementary Figures





**Fig. S1.** **Evaluation of printing fidelity across filament types.** Representative microstructures fabricated using (A) PP, (B) PLA, and (C) nylon filaments are shown, with a scale bar of 200 µm. Among the tested channel architectures, those fabricated with nylon exhibited superior structural fidelity, maintaining precise geometry without ceiling sagging or border deformation. PLA, while easier to process, falls short in mechanical stability, and PP suffers from pronounced warping and adhesion deficiencies due to its low density, demanding specialized adhesives and extensive supports. Nylon therefore represents the most balanced choice, achieving high structural accuracy while maintaining efficient and reliable manufacturability.





**Fig. S2.** **The selection of finger tight screw and design** **optimization of input/outlet ports**. Optical microscopy image of the (A) finger tight screw, (B) outlet tubing at 5x magnification and (C) the SketchUp design of the ports connection, with a reference bar of 0.5 mm.

Design optimization of the input/outlet ports was guided by the need to minimize turbulence and prevent droplet coalescence during egress. Standard finger-tight screw fittings, though convenient, featured large diameters (~2.1 mm) that lowered exit velocity, leading to droplet retention and merging. By threading the tubing through the fitting and embedding it within a wider sleeve, the effective opening was reduced to ~0.8 mm, with additional narrowing by the terminal tubing (~0.42 mm outer diameter). This geometry adjustment decreased the Reynolds number, enabling stable droplet transport and consistent release.





**Fig. S3. Design of different channels and microfluidic bases.** Examples of (A) cross junction channel, (B) curved channel, (C) regular hexagonal straight channel, (D) T-junction base and (E) flow-focusing junction bas that could be used in microfluidic device designing.





**Fig. S4. Optimization of dispersed-phase channel depth for 265 µm width.** The influence of depth on the structural fidelity and functional performance of 265 µm-wide dispersed-phase channels was investigated by fabricating variants with depths of 0.1 mm (A), 0.2 mm (B), 0.3 mm (C), and 0.4 mm (D). Scale bar: 200 µm.





**Fig. S5. Optical microscopy characterization of critical regions in flow-focusing junctions.** Optical microscopy of the junction snippet in Fig.3A at different channel lengths of (A) 0.5, (B) 1.0, (C) 1.5, and (D) 2.0 mm. At each channel length, the outlet channel, junction and dispersed-phase channel are focused on. The reference bar is 200 µm.

Junction segments were fabricated under identical printing conditions as the full microfluidic devices to visualize potential structural issues. High-resolution optical microscopy focused on three regions: (i) the outlet channel, capturing droplet egress, (ii) the junction interior, where sharp corners are susceptible to overflow-induced turbulence, and (iii) the dispersed-phase inlet channel, confirming unobstructed fluid entry. This targeted analysis enables precise design refinement to enhance droplet formation stability and device reliability.





**Fig. S6.** **Geometrical fidelity assessment of FDM-printed Microfluidic Channels.** The fidelity of microfluidic channel geometries was assessed by comparing computer-aided designs with their printed counterparts, focusing on (A) square, (B) diamond, and (C) hexagonal cross-sections, with a scale bar of 200 µm.

Analysis reveals intrinsic limitations of FDM in reproducing ideal geometries, particularly due to ceiling drooping during fabrication, impairing flow characteristics. Among the tested profiles, the hexagonal cross-section demonstrated superior structural preservation. The hexagonal profile mitigates this effect by employing a height 15% greater than its width (100 % / cos 30°), which stabilizes the channel ceiling and preserves flow uniformity. Such geometrical compensation strategies provide a practical pathway to overcoming inherent resolution constraints in polymer extrusion printing.


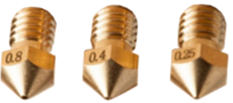


**Fig. S7. Different nozzles with different orifice diameter.**

In fused deposition modeling (FDM), nozzle orifice diameters typically range from 0.1 to 1 mm, with 0.4 mm being the standard in commercial printers. This parameter directly governs key resolution-related variables, including layer thickness, raster width, air gap, and printing speed.^[3,4]^ Larger orifices extrude greater material volumes, enabling faster deposition but at the expense of resolution, whereas smaller orifices (<0.4 mm) enable finer feature definition with minimal layer artifacts, albeit at reduced throughput. Importantly, the nominal orifice diameter refers to the internal tip aperture, independent of the overall nozzle size. Comparative studies have demonstrated that smaller diameters (0.2–0.25 mm) increase printing time and induce higher pressure drops, both of which influence extrusion stability and dimensional fidelity.^[5]^ In this work, we identified 0.25 mm as the optimal compromise between resolution, printing efficiency, and extrusion consistency, delivering superior structural precision while maintaining acceptable fabrication speed.





**Fig. S8.** **Channel printing optimization through** **obj. to G code conversion analysis.**

A comprehensive understanding of how printing parameters influence final channel architecture requires examining the fundamental process of shape translation from the digital object file to the generated G-code. (A) 350 and (C) 1045 µm end cross-section of the SketchUp drawn hexagon channel (green hexagon) overlapped with the Cura layers to be printed (orange). The printed channel of the (B) 350 and (D) 1045 µm width hexagon channels, with a reference bar of 200 µm.





Fig. S9. The correlation assessment between designed and printed widths for both the channel and junction. (A) Illustration of the difference between fabricated and designed width, with examples at (B) 435, (C) 615, (D) 790, and (E) 955 µm, were also shown in Fig. 3E in the main manuscript. The reference bar is 200 µm.

The fabrication process was evaluated with emphasis on both the main channel width and the junction width, defined as the hexagonal segment at the channel terminus adjoining the junction. Measured values for both parameters were plotted against the designed dimensions (Fig. S8A), with representative micrographs shown in Fig. S8B–E. Linear regression analysis revealed that the channel width followed y=0.95x−185, indicating an average undersizing of 185 µm relative to the design. In contrast, the junction width followed y=1.00x+24, averaging 24 µm larger than intended.

**Fig. S10. Assessment of coatings affinity for stable droplet formation in microfluidic devices.** Incorporation of diverse hydrophobic and lipophobic fluorinated modifiers into epoxy resin significantly modulates the contact angles of water, cyclohexane, and octane. All measurements were conducted in triplicate, with corresponding standard deviations reported to ensure statistical rigor.

***
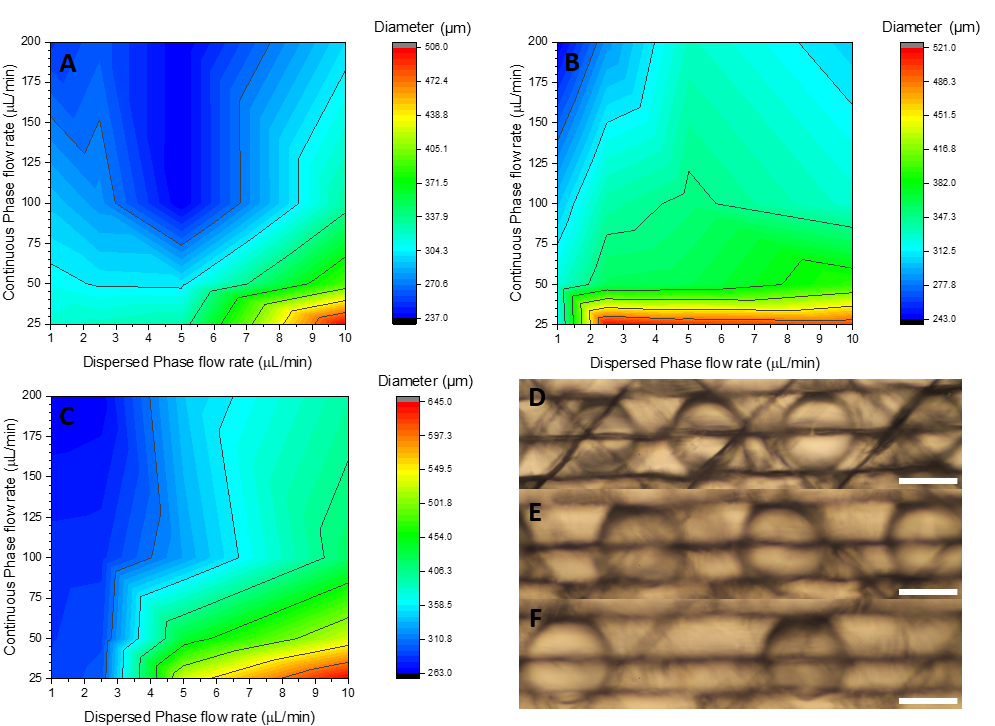
***

Fig. S11. Droplet generation performance of T-junction microfluidic devices 175 µm dispersed-phase channel. The droplet size from the odd layer devices with a dispersed-phase flow rates from 1 to 10 µL/min and continuous-phase flow rate from 25 to 200 µL/min in T-junction microfluidic. The odd layer devices had a dispersed-regular hexagon width of 175 µm, whilst the continuous-phase had a regular hexagon width of (A) 530, (B) 700 and (C) 880 µm. At 5 µL/min dispersed-phase to 100 µL/min continuous-phase droplets are shown for halved regular hexagon width of (D) 530, (E) 700 and (F) 880 µm. The reference bar is 300 µm.


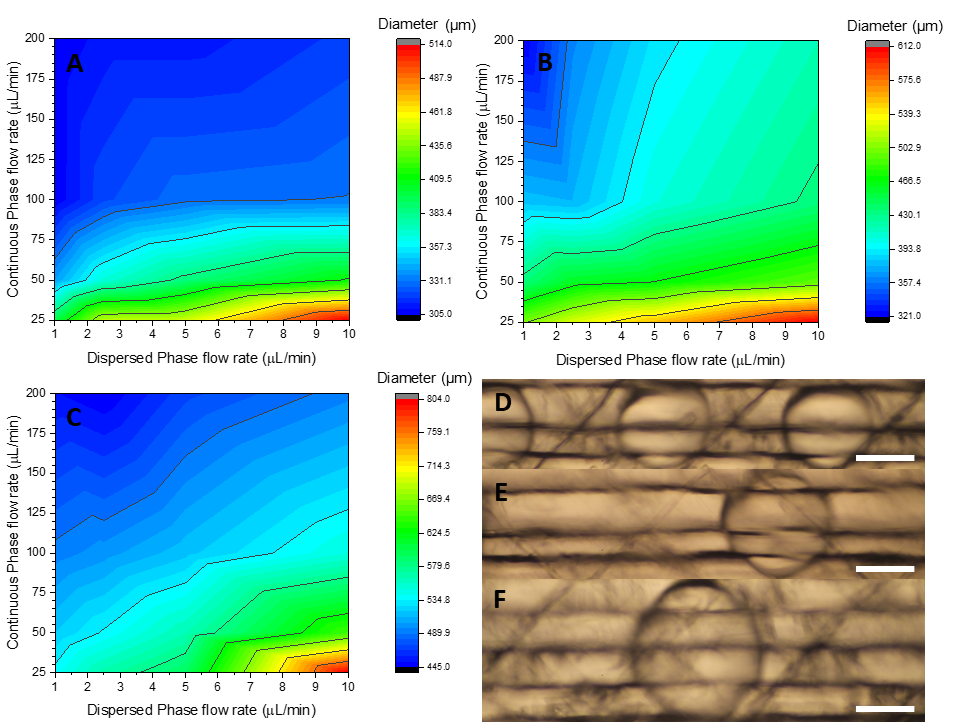


**Fig. S12. Droplet generation performance of T-junction microfluidic devices with a 265 µm dispersed-phase channel*.*** The droplet size from the odd layer devices with a dispersed-phase flow rates from 1 to 10 µL/min and continuous-phase flow rate from 25 to 200 µL/min in T-junction microfluidic. The odd layer devices had a dispersed-regular hexagon width of 265 µm, whilst the continuous-phase had a regular hexagon width of (A) 620, (B) 790 and (C) 960 µm. At 5 µL/min dispersed-phase to 100 µL/min continuous-phase droplets are shown for regular hexagon width of (D) 620, (E) 790 and (F) 960 µm. The reference bar is 300 µm.

In T-junction devices, minimum droplet size (~263 µm) was achieved with 175 µm dispersed-phase channel, as high flow rates limited wall contact and breakup depended primarily on junction-edge sharpness and pressure differential. At lower flow rates, wall interaction altered droplet formation, and near pressure balance caused junction flooding, producing droplets >0.5 mm. Reducing the dispersed-phase channel to 175 µm did not improve droplet size or match flow-focusing junction performance, but increased clogging risk. These findings show that geometric downscaling alone does not enhance T-junction efficiency, reinforcing the superiority of flow-focusing junctions for smaller, more uniform droplet generation.

**Fig. S13. The complex viscosity of the alginate solutions as a function of shearing rate.** Rheological analysis of alginate solutions (1-5 wt%) at ambient temperature revealed a pronounced increase in viscosity with concentration, directly correlating with larger droplet sizes during emulsification. This relationship underscores the critical influence of solution viscosity on droplet formation dynamics.





**Fig. S14. Controllable generation of W/O droplets with different dispersed and continuous flow rates.** The dispersed phase consist of 1.5 wt% alginate solution. Optical microscopy images capture droplet formation at dispersed phase flow rate fixed at 5 μL/min and continuous phase flow rates of (A) 50 μL/min, (B) 100 μL/min, and (C) 150 μL/min. Corresponding droplet size distributions are presented in panels (D-F). Scale bar: 200 µm.





**Fig. S15. Formation of irregular alginate microgels with distinct morphologies.** Alginate microgel particles exhibited diverse and uncontrolled morphologies, including (A-B) characteristic mushroom-shaped structures and (C) irregular forms featuring pronounced tail-like extensions.

In the absence of pre-gelation within the microfluidic channel, droplets undergo deformation during external gelation due to the combined effects of gravity and viscous forces. As droplets descend through the gelation bath, they experience competing forces including gravity, buoyancy, interfacial tension, and viscous drag. Upon exiting the collection tubing, droplets strive to regain a spherical shape to minimize interfacial free energy. The initial droplets rapidly enter the Newtonian gelation bath, preserving their prolate shape. In this regime, the droplet tail elongates progressively due to the interplay between viscous and elastic forces governing the gelation kinetics.





**Fig. S16. Enzyme activity and stability.** Evolution of pH by alternation of (A) urease and (D) esterase in free or encapsulated conditions. Influence of pH on the enzymatic activity of (B) urease and (E) esterase in free or encapsulated conditions. Residual activity of (C) immobilized and free urease, (F) immobilized and free esterase as a function of time.





**Fig. S17. Reversible pH-responsive behavior of UMCs.** (A) Comparative hydrodynamic diameter distributions of alginate microspheres and UMCs. (B) Cyclic swelling–shrinking responses of UMCs under alternating pH conditions (pH 8.0 to 4.0) over six consecutive cycles, demonstrating robust reversibility. Standard deviations are shown as error bars.





**Fig. S18. pH modulation and self-regulating capabilities of UMCs.** Self-regulating pH-responsive behavior of UMCs was assessed by modulating urease–urea reaction parameters. (A) Effect of varying urease concentration at a fixed urea concentration (100 mM). (B) Effect of urea concentration at a constant urease concentration (5 mg/mL). Data represent triplicate measurements with standard deviations.





**Fig. S19.** **Reversible pH-responsive behavior of EMCs.** (A) Comparative hydrodynamic diameter distributions of alginate microspheres and EMCs. (B) Cyclic swelling–shrinking responses of EMCs under alternating pH conditions (pH 4.0 to 8.0) over six consecutive cycles, demonstrating robust reversibility. Standard deviations are shown as error bars.





**Fig. S20. pH modulation and self-regulating capabilities of EMCs.** Self-regulating pH-responsive behavior of EMCs was assessed by modulating esterase–EA reaction parameters. (A) Effect of varying esterase concentration at a fixed urea concentration (100 mM). (B) Effect of EA concentration at a constant esterase concentration (8 mg/mL). Data represent triplicate measurements with standard deviations.





**Fig. S21. Evaluation of pH-induced negative feedback-loop of microcapsules by addition of free enzymes.** (A) Base-mediated negative feedback in UMCs was assessed by adding 1 mL of 1 wt% free urease at a steady-state pH (~7.5). The subsequent pH increase confirmed that regulation arises from shell permeability modulation rather than substrate depletion. (B) Acid-mediated negative feedback in EMCs was evaluated by introducing 1 mL of 1 wt% free esterase at a steady-state pH (~6).

The observed pH decrease validated acid-triggered feedback via shell permeability. Plateau values were consistent with pH modulation trends shown in Fig. S18B and S20B. All pH measurements are reported as the mean ± SD of ≥3 independent replicates.


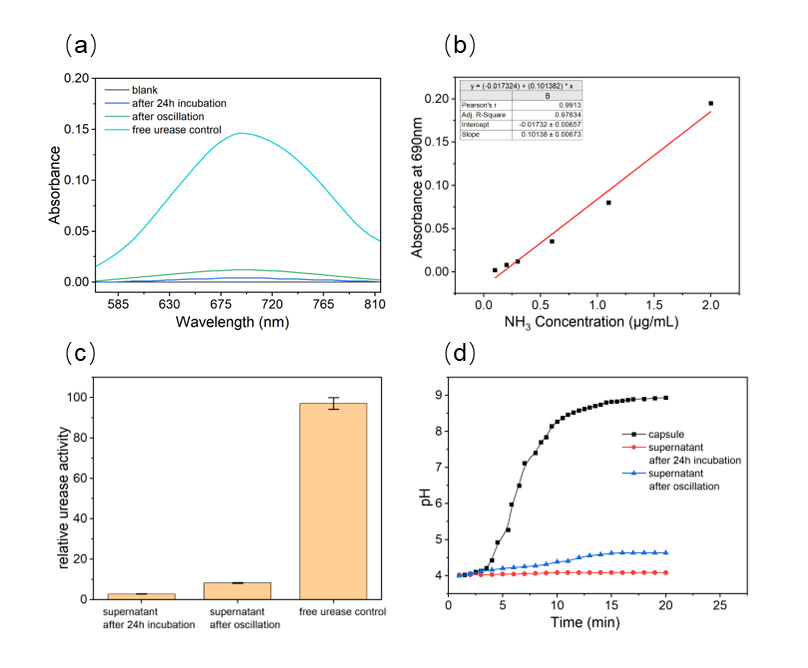


**Figure S22. The quantitative control enzyme leakage experiments of UMCs system.** (a) The UV-vis of supernatants after capsule preparation and pH oscillation experiments. (b) The calibration curve of NH_3_ concentration. (c) the relative urease activity of supernatants compared to free urease. (d) the contribution of leaked urease to the observed regulation behavior.

The supernatant was analyzed for ammonium content by a reagent kit (Spectroquant® Ammonium, Merck) based on the indophenol blue method. Quantitative ammonium assays demonstrated negligible urease activity in the post-preparation supernatant, confirming the great structural stability and enzyme retention capacity of the as-prepared UMCs. There are weak but detectable urease activity was observed in the supernatant after pH oscillation experiments, with the relative activity remaining below 10% (Figure S22c). Moreover, under identical urea-containing test conditions, neither the post-preparation nor post-oscillation supernatant reproduced the pH increase result observed in the UMCs system (Figure S22d). These results demonstrate that enzyme leakage from UMCs is negligible throughout preparation and pH oscillation. Also, any leaked free urease contributes marginally to the pH regulation behavior. The observed pH oscillation and regulation behavior are dominated exclusively by the catalytic activity of urease encapsulated within the pH-responsive microcapsules.


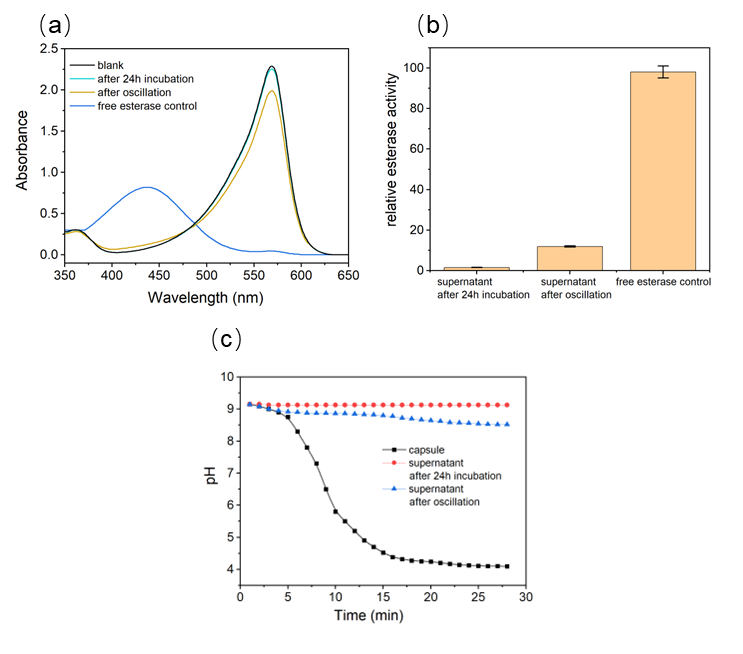


**Figure S23. The quantitative control enzyme leakage experiments of EMCs system**. (a) The UV-vis of supernatants after capsule preparation and during oscillation experiments. (b) the relative urease activity of supernatants compared to free esterase. (c) the contribution of leaked urease to the observed regulation behavior.

The UMCs and EMCs adopt the same preparation strategy, the very low supernatant enzyme activity in the urease system supports that the capsule structure has good enzyme retention capacity. Considering the molecular characteristics of different enzymes and their interactions with the shell may vary, we further conducted specialized relative enzyme activity and control experiments on the supernatant of EMCs. For EMCs, the relative esterase activity in the collected supernatants was evaluated by a phenol-red-based pH-shift assay using ethyl acetate as substrate, and the absorbance at 560 nm was recorded.

The free esterase positive control exhibited a dramatic decrease at 560 nm, indicating efficient ethyl acetate hydrolysis and rapid acid generation. In contrast, the post-preparation supernatant showed almost no absorbance change nearly identical to the blank control, while the supernatant after pH oscillation displayed only a marginal absorbance decrease (Figure S23a). The relative enzyme activity revealed that the esterase activity in the post-preparation supernatant was close to the background level, and the relative activity in the post-oscillation supernatant remained at a very low level (Figure S23b), confirming the excellent enzyme retention capacity of EMCs. As same as UMCs, the pH regulation experiments showed that the leaked esterase in the supernatants makes limited contribution to the pH regulation behavior (Figure S23c).





**Fig. S24. pH stabilization by varying UMCs/EMCs ratios in response to external acid or base generation**. The pH-regulating performance of homeostatic microcapsules was systematically investigated under acid- and base-generating conditions. By tuning the UMC/EMC ratio, distinct self-regulation behaviors emerged, yielding characteristic pH trajectories and stabilization points. (A) Acid-triggered pH modulation for varying capsule ratios. (B) Alkali-triggered pH modulation for varying capsule ratios.





**Fig. S25. pH stabilization by varying Urea/EA ratios in response to external acid or base generation**. The bidirectional homeostatic control of solution pH can be finely tuned by adjusting the ratio of urea and EA-loaded medium (Urea/EA). Under both acid- and base-generating stimuli, capsule ensembles exhibited distinct feedback-regulated pH trajectories and stabilized at characteristic equilibrium values. (A) Acid-induced regulation profiles across different Urea/EA ratios. (B) Base-induced regulation profiles across different Urea/EA ratios.


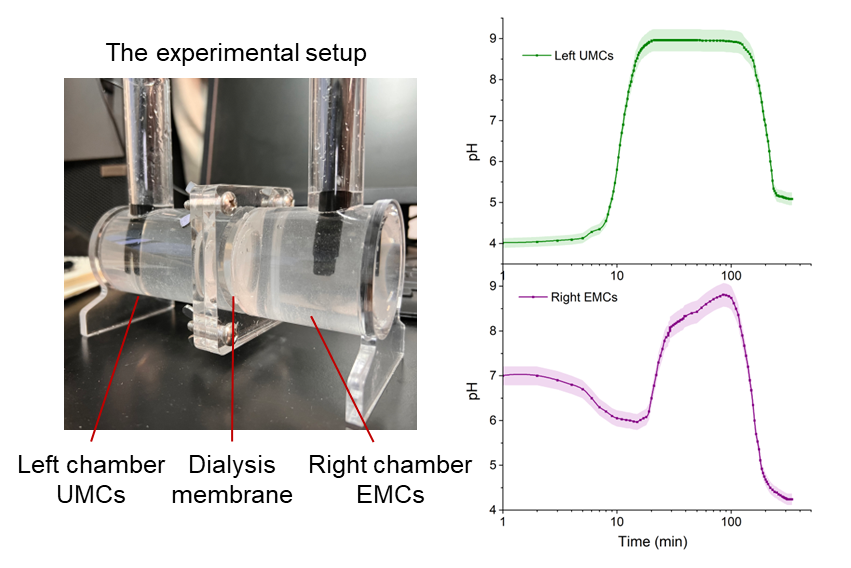


**Figure S26.** (A) The experimental setup that physically separate the two capsule populations while allowing diffusion of small molecules. (B) the pH regulation behavior by evidence of localized interactions.


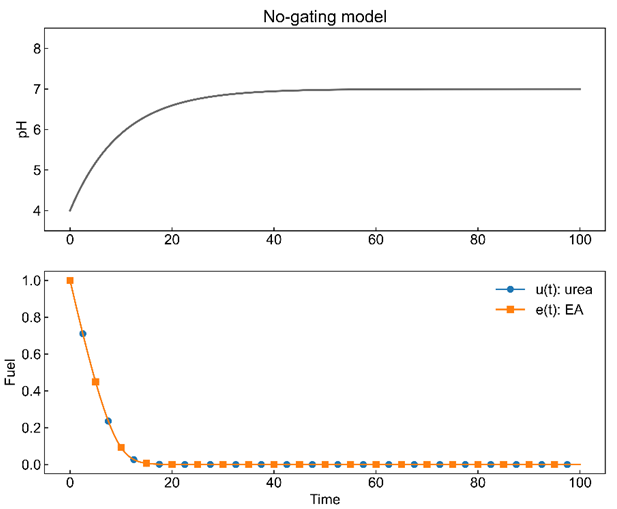


**Fig. S27.** The no-gating control simulation. In the absence of pH-responsive shells, the system pH only rises to a near-neutral plateau with running out of fuels, without oscillatory behavior.

A single variable effective alkalinity *x*(t) was introduced, when *x* > 0 the system is more alkaline. pH is obtained from x through a simple mapping:

$$pH\left( t \right)=pH_{0}+\lambda x\left( t \right)$$

Where pH_0_ is the reference pH, in our model we set pH_0_ = 7. 𝞴 converts effective alkalinity into pH change.

*R*_U_ and *R*_E_ are the effective reaction rates of the UMC (alkalization) branch and EMC (acidification) branch respectively. We first consider the no-gating model:

$$R_{U}=k_{U}\frac{u}{K_{U}+u}$$

$$R_{E}=k_{E}\frac{e}{K_{E}+u}$$

Where *u* and *e* are the amounts of urea and ethyl acetate, *k*_U_ and *k*_E_ are effective kinetic rate constants respectively, and K_U_, K_E_ are saturation constants that determine how strongly the reaction rates depend on the available fuel concentration.

We consider the bulk urea and EA reservoirs decay with depletion scaling factors ε_U_ and ε_E_, thus the rates of consuming are:

$$\frac{du}{dt}= -\varepsilon_{U}R_{U}$$

$$\frac{de}{dt}= -\varepsilon_{E}R_{E}$$

They contribute to the effective alkalinity dynamics according to

$$\frac{dx}{dt}= {\beta_{U}R}_{U}-{\beta_{E}R}_{E}-kx$$

Where 𝛽_U_ and 𝛽_E_ are conversion coefficients that control the contribution of UMC and EMC reaction rates to net alkalization and acidification. *k* is relaxation rate constant for *x*, deriving the system back toward *x* = 0.


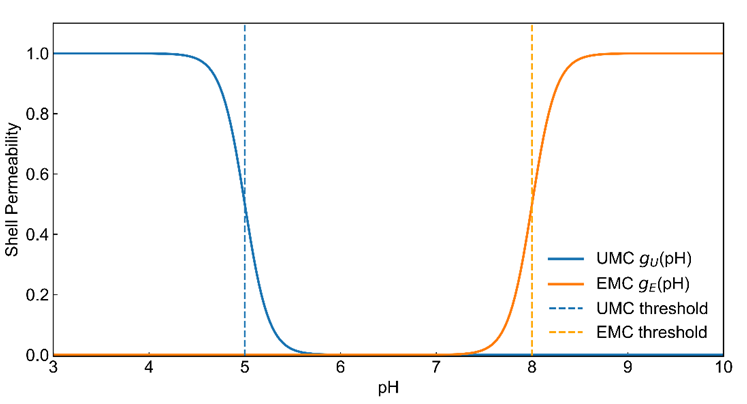


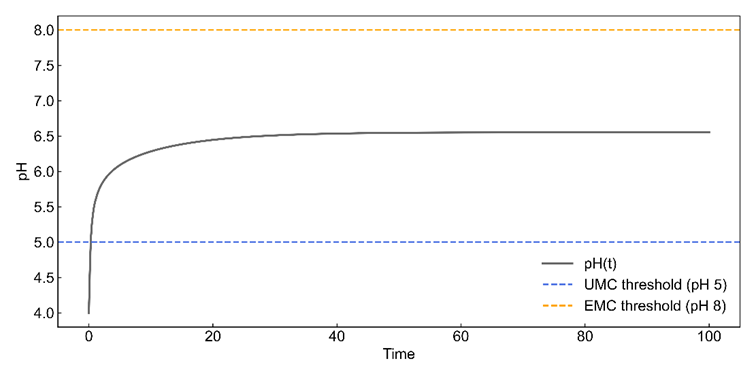


**Fig. S28.** The shell permeability change of the capsules. UMC becomes permeable below pH 5, and EMC becomes permeable above pH 8.

Two target sigmoid functions for the pH-dependent shell permeability *g*_U_ and *g*_E_:

$$g_{U}\left( \mathrm{pH} \right)= \frac{1}{1+\exp[s_{U}(\mathrm{pH}-\mathrm{pH}_{U,on})]}$$

$$g_{E}\left( \mathrm{pH} \right)= \frac{1}{1+\exp[{-s}_{E}(\mathrm{pH}-\mathrm{pH}_{E,on})]}$$

Here, pH_U,on_ and $\mathrm{pH}_{E,on}$ are characteristic pH at which they are half-switched (*g* = 0.5), *s*_U_ and *s*_E_ determine the steepness of the sigmoidal transitions, how fast the shell state changes with pH.


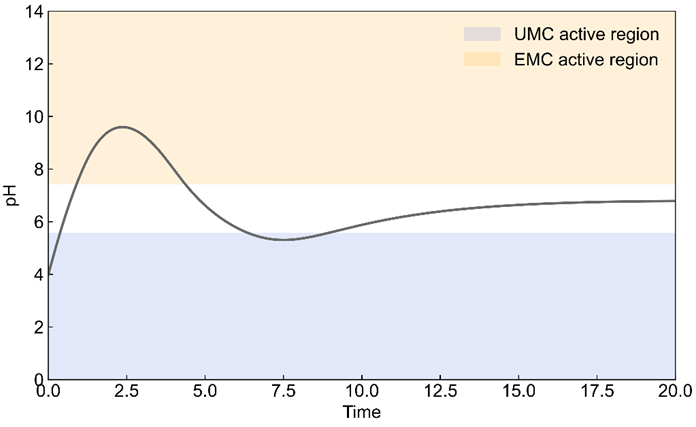


**Fig. S29.** The delayed response model results. Starting from an initial pH of 4, the simulated pH rises into the EMC active region and then falls back into the UMC active region.

Incorporating the shell permeability, the effective rates become:

$$R_{U}=k_{U}g_{U}\left( \mathrm{pH} \right) \frac{u}{K_{U}+u}$$

$$R_{E}=k_{E} g_{E}\left( \mathrm{pH} \right)\frac{e}{K_{E}+u}$$

Further changes the rates of consuming $\frac{dU}{dt}$, $\frac{de}{dt}$, and effective alkalinity $\frac{dx}{dt}$. Under this instantaneous switch-on/switch-off regime, the system pH increases abruptly after passing pH5. We decrease *s*_U_ and *s*_E_ to consider the possibility that shell permeability changes continuously, such that the shells undergo gradual rather than abrupt opening and closing, the pH response is continuous but still not enough to trigger EMC.

Considering the shell response has a delay effect, we introduced *τ*_U_ and *τ*_E_ as characteristic response time of shells, giving actual shell permeability states *q*_U_(t) and *q*_E_(t):

$$\frac{dq_{U}}{dt}= \frac{g_{U}\left( pH \right)-q_{U}}{\tau_{U}}$$

$$\frac{dq_{E}}{dt}= \frac{g_{E}\left( pH \right)-q_{E}}{\tau_{E}}$$

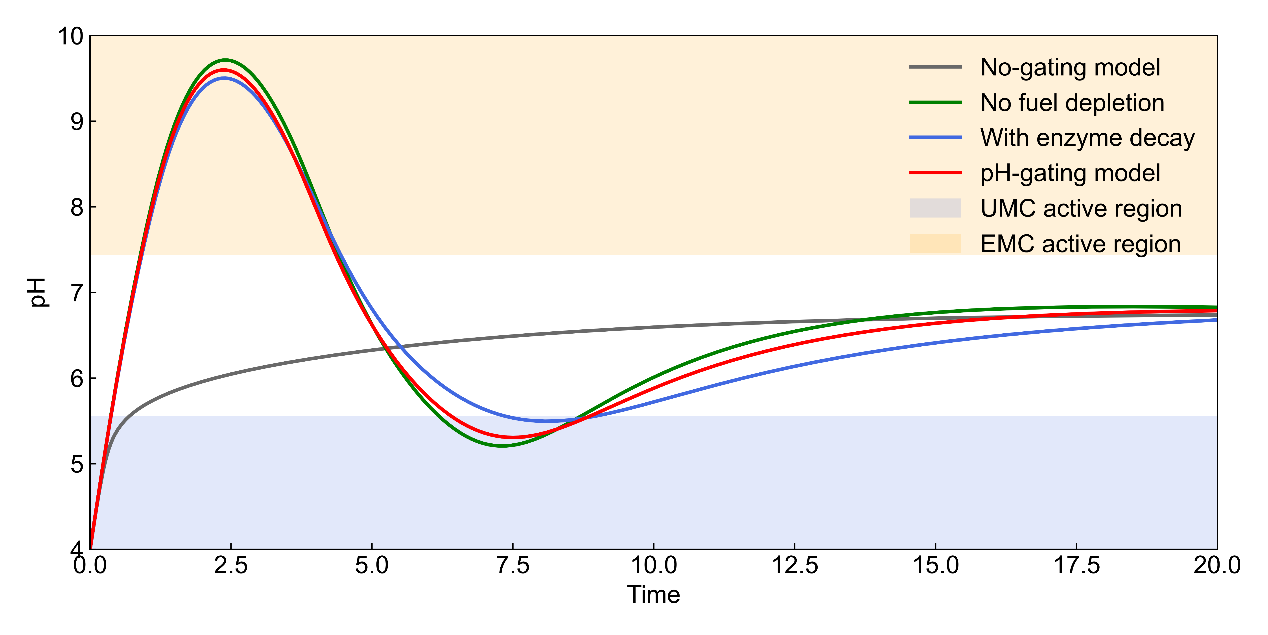


**Fig. S30.** The single-factor control simulations. The quantitative model separates the contributions of different factors to the oscillatory behavior.

**Table. S1. Calculating the SketchUp width for well printed regular hexagon channels with an even number of layers**

| Layers | Height (µm) | Theoretical Width (µm) | SketchUp Width (µm) |
| --- | --- | --- | --- |
| 2 | 100 | 86.6 | 90 |
| 4 | 300 | 259.8 | 265 |
| 6 | 500 | 433.0 | 435 |
| 8 | 700 | 606.2 | 615 |
| 10 | 900 | 779.4 | 790 |
| 12 | 1100 | 952.6 | 955 |
| 14 | 1300 | 1125.8 | 1130 |

**Table. S2. Calculating the SketchUp width for well printed regular hexagon channels with an odd number of layers**

| Layers | Height (µm) | Theoretical Width (µm) | SketchUp Width (µm) |
| --- | --- | --- | --- |
| 3 | 200 | 173.2 | 175 |
| 5 | 400 | 346.4 | 350 |
| 7 | 600 | 519.6 | 525 |
| 9 | 800 | 692.8 | 700 |
| 11 | 1000 | 866.0 | 870 |
| 13 | 1200 | 1039.2 | 1045 |

**Table S3. The corresponding capillary number (Ca) and Weber number (We)**

| Regime | Q_d_ (μL/min) | Q_c_ (μL/min) | Q_c_/Q_d_ | $v_{c}$ (mm/s) | Ca | We |
| --- | --- | --- | --- | --- | --- | --- |
| Dripping | 5 | 100 | 20 | 5.80 | 5.56◊10^-3^ | 5.89◊10^-4^ |

*Capillary number (*Ca*):*

$$C_{a}=\frac{\mu_{c}v_{c}}{\gamma}$$

where $\mu_{c}$ is the dynamic viscosity of the continuous phase (corn oil), $v_{c}$ is the characteristic flow velocity of the continuous phase in the main channel, and $\gamma$ is the interfacial tension between the dispersed and continuous phases.

*Weber number (*We*):*

$$W_{e}=\frac{\rho_{c}v_{c}^{2}D_{h}}{\gamma}$$

where $\rho_{c}$ is the density of the continuous phase, and $D_{h}$ is the hydraulic diameter of the continuous phase channel.

**Table S4. The corresponding parameter**

| parameter | value |
| --- | --- |
| $\mu_{c}$ | 30.5 mPa·s |
| $v_{c}$ | 5.80 mm/s |
| $\rho_{c}$ | 920 kg·m^-3^ |
| $\gamma$ | 31.8 mN/m |
| $D_{h}$ | 605 μm |

The cross-sectional area of continuous phase channel is calculated as follow:

$$A=\frac{\pi D_{h}^{2}}{4}=2.87\times{10}^{-7}m^{2}$$

**Table S5. The device consumable cost breakdown**

| **Consumable Item** | **Commercial**  **Cost** | **Consumption**  **per Device** | **Cost per**  **Device (USD)** |
| --- | --- | --- | --- |
| Nylon Filament | $60.00/kg | 10-15 g | $0.6-0.9 |
| Epoxy Resin | $100.00/kg | 2-5 g | $0.2-0.5 |
| Fluorinated Hydrophobic Modifier | $200.00/L | 1-3 mL | $0.2-0.6 |
| PTFE Tubing (1/16" OD) | $0.80/m | ~2 m | $1.6 |
| PEEK Lock Fittings | $1.45/piece | 4 pieces | $5.8 |
| Isopropyl Alcohol | $110.00/L | 2-4 mL | $0.22-0.44 |
| **Total Consumable Cost** | $8.62-9.84 | | |

**Table S6. The coefficient of variation comparison**

| Fabrication materials | CV (%) | Ref in SI |
| --- | --- | --- |
| quartz glass | 3-8% | 6 |
| planar quartz glass | 1.6-5.3% | 7 |
| quartz glass | ＜5% | 8 |
| glass capillary tube | 1.3% | 9 |
| glass capillary | 3.88% | 10 |
| PDMS | 10% | 11 |
| PDMS | ~3% | 12 |
| PDMS | ~3% | 13 |
| PDMS | ~1.5% | 14 |
| 3D-printed Prusa resin | ~3% | 15 |
| TangoPlus FLX930, rubber | ~5% | 16 |
| acrylate clear resin | ~4% | 17 |
| Nylon | 1-3% | This work |

**Movie S1.**

Controlled generation of uniform droplets within a flow-focusing junction (dispersed phase: 5 μL/min, continuous phase: 50 μL/min).

**Movie S2.**

Dynamic transport of generated droplets from Movie S1 along a co-flow channel under a continuous-phase flow rate of 50 μL/min.

**Movie S3.**

Controlled generation of uniform droplets within a flow-focusing junction (dispersed phase: 5 μL/min, continuous phase: 100 μL/min).

**Movie S4.**

Dynamic transport of generated droplets from Movie S1 along a co-flow channel under a continuous-phase flow rate of 100 μL/min.

**Movie S5.**

Droplets generated in T junction, with a dispersed-phase flow rate of 5 μL/min, and a continuous-phase flow rate of 100 μL/min.

**Movie S6.**

Transition of generated droplets from a narrow junction channel into a wider collection channel at a continuous-phase flow rate of 100 μL/min.

**References**

1. M. Fidaleo, R. Lavecchia, Kinetic Study of Enzymatic Urea Hydrolysis in the pH Range 4-9. *Chem. Biochem. Eng. Q*. **2003**, 17, 311.

2. L. Ramnath, B. Sithole, R. Govinden, Identification of lipolytic enzymes isolated from bacteria indigenous to Eucalyptus wood species for application in the pulping industry. *Biotechnol Rep*. **2017**, 15, 114.

3. A. Dey, N. Yodo, A Systematic Survey of FDM Process Parameter Optimization and Their Influence on Part Characteristics. *J. Manuf. Mater. Process*. **2019**, 3, 64.

4. O. A. Mohamed, S. H. Masood, J. L. Bhowmik, Optimization of fused deposition modeling process parameters: a review of current research and future prospects. *Adv Manuf.* **2015**, 3, 42.

5. G. C. Nzebuka, C. O. Ufodike, A. M. Rahman, C. M. Gwynn, M. F. Ahmed, Numerical modeling of the effect of nozzle diameter and heat flux on the polymer flow in fused filament fabrication. *J Manuf Process.* **2022**, 82, 585.

6. Y Liu, N Tottori, T Nisisako. Microfluidic synthesis of highly spherical calcium alginate hydrogels based on external gelation using an emulsion reactant. *Sensors and Actuators B: Chemical*, **2019**, 283, 802-809.

7. T Nisisako, T Hatsuzawa. Microfluidic fabrication of oil-filled polymeric microcapsules with independently controllable size and shell thickness via Janus to core–shell evolution of biphasic droplets. *Sensors and Actuators B: Chemical*, **2016**, 223, 209-216.

8. D. R. Link, S. L. Anna, D. A. Weitz and H. A. Stone, Geometrically mediated breakup of drops in microfluidic devices. *Phys. Rev. Lett.*, **2004**, 92, 054503.

9. A. S. Utada, E. Lorenceau, D. R. Link, P. D. Kaplan, H. A. Stone and D. A. Weitz, Monodisperse double emulsions generated from a microcapillary device. *Science*, **2005**, 308 , 537-541.

10. W. Ma, C. Mou, S. Chen, Y. Li and H. Deng, A mild method for encapsulation of citral in monodispersed alginate microcapsules. *Polymer*, **2022**, 14 , 1165.

11. N Tottori, S Choi, T Nisisako. Production of monodisperse oil-in-water droplets and polymeric microspheres below 20 μm using a PDMS-based step emulsification device. *Micromachines*, **2025**, 16, 132.

12. K.Sun, J.Zeng, Y.Liu, Z.Zhou, J.Chen, J.Chen, X.Huang, F.Gao, X.Wang, X.Zhang, X.Wang, S.Eeltink, B.Zhang, Microfluidic precision manufacture of high performance liquid chromatographic microspheres *Angew. Chem. Int. Ed*. **2025**, 64, e202418642.

13. C.H.Y. Chung; B. Cui; R. Song; X. Liu; X. Xu; S. Yao. Scalable production of monodisperse functional microspheres by multilayer parallelization of high aspect ratio microfluidic channels. *Micromachines*, **2019**, 10, 592.

14. Z Lian, Y Ren, J He, et al. Microfluidic fabrication of porous polydimethylsiloxane microparticles for the treatment of toluene-contaminated water. *Microfluidics and Nanofluidics*, **2018**, 22, 145.

15. A. B. Zia , J. Farrell and I. G. Foulds, Automated dynamic inlet microfluidics system: 3D printer adaptation for cost-effective, low volume, on-demand multi-analyte droplet generator. *Lab Chip*, **2024**, 24 , 3015-3026.

16. Q Ji, J M Zhang, Y Liu, et al. A modular microfluidic device via multimaterial 3D printing for emulsion generation. *Scientific reports*, **2018**, 8, 4791.

17. Y H Hwang, T Um, G N Ahn, et al. Robust and scalable production of emulsion-templated microparticles in 3D-printed milli-fluidic device. C*hemical Engineering Journal*, **2022**, 431, 133998.
